# Supplementary material for: Regulation of tension-dependent localization of LATS1 and LATS2 to adherens junctions
Source: PLoS One. 2026 Feb 2;21(2):e0342107. doi: 10.1371/journal.pone.0342107 (PMC12863670; doi:10.1371/journal.pone.0342107)
Supplement: S1 Fig — (PDF) [file pone.0342107.s001.pdf]

Figure 1B

IP-FLAG_LIMD1 (GFP)

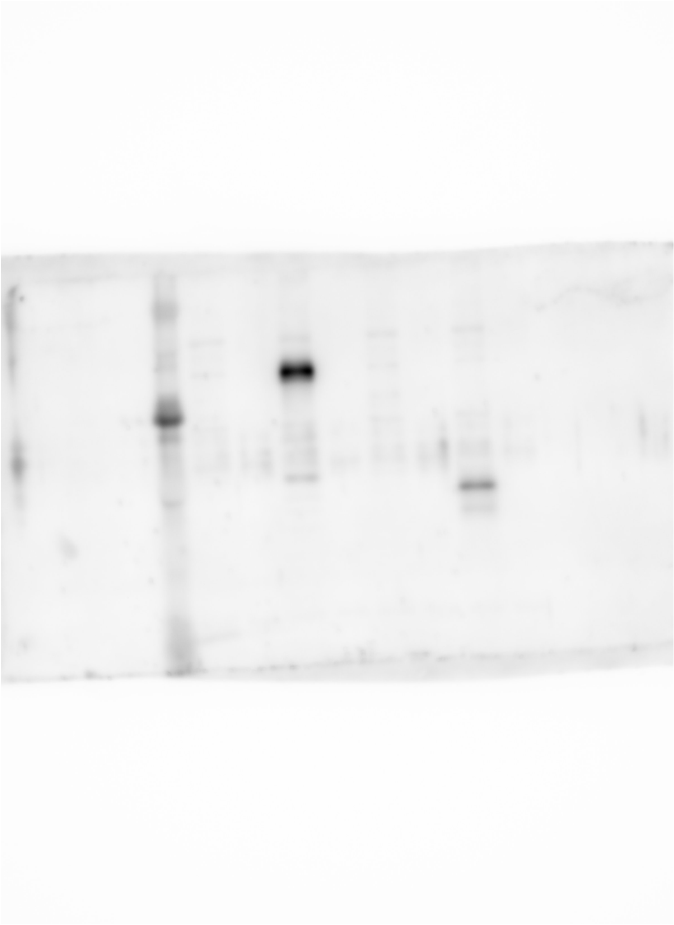

.TIF file

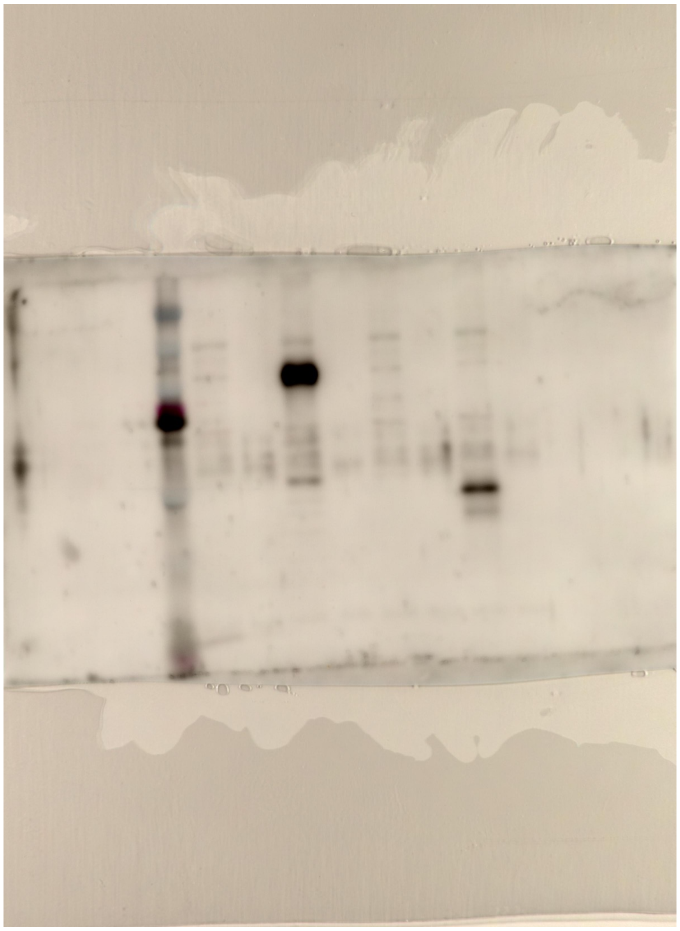

JPG file

Figure 1B

IP-FLAG_LATS2

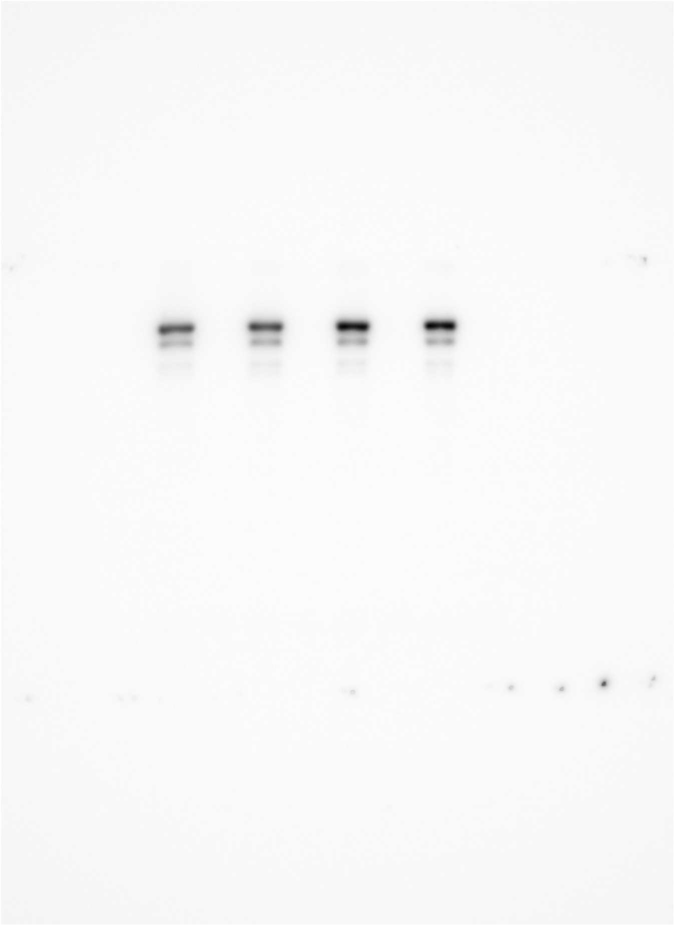

.TIF file

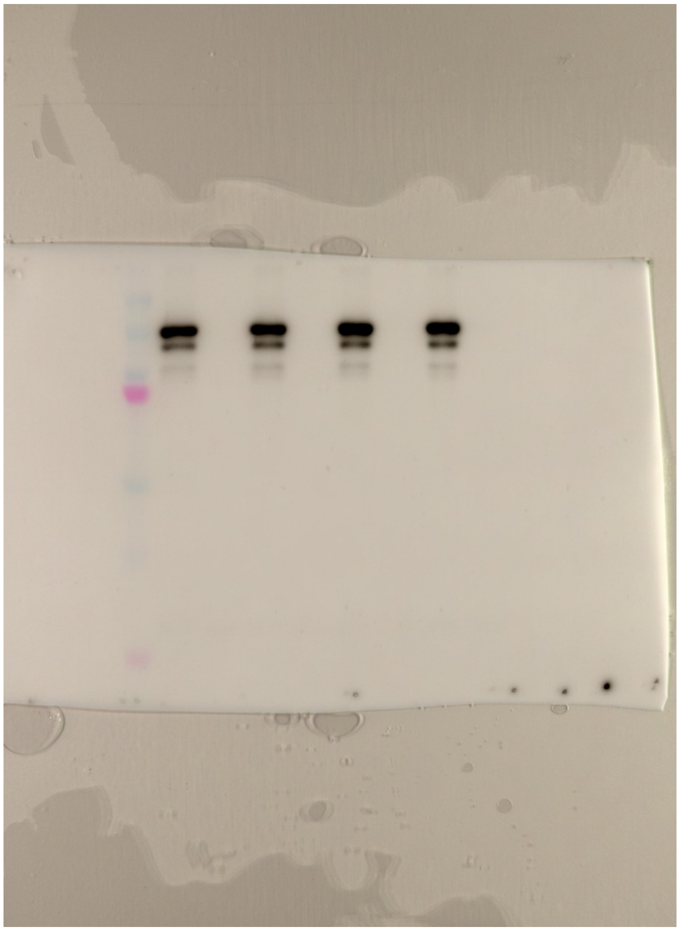

JPG file

Figure 1B

Input_LIMD1 (GFP)

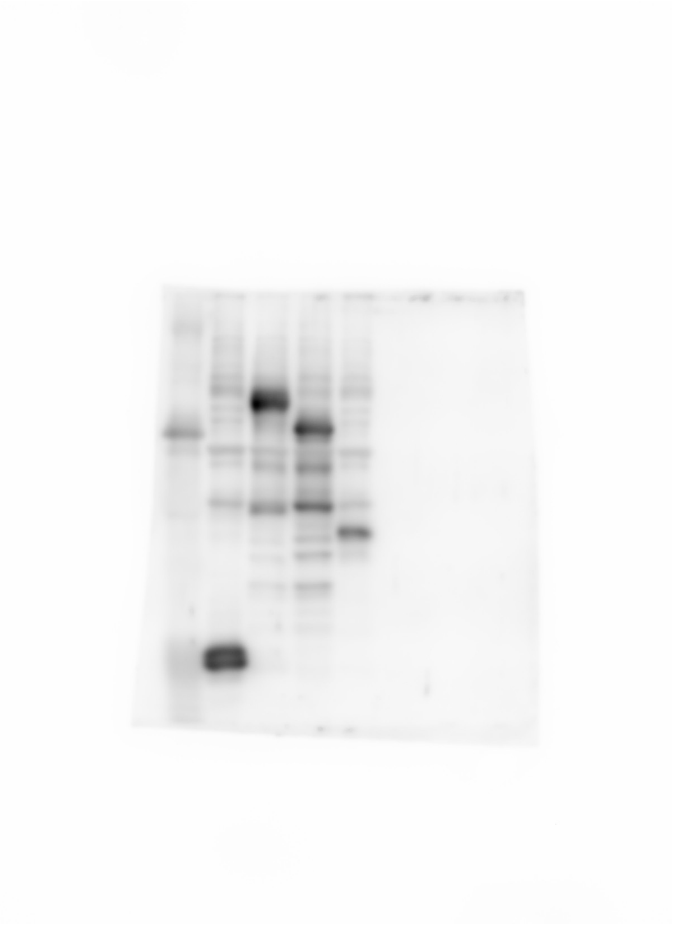

.TIF file

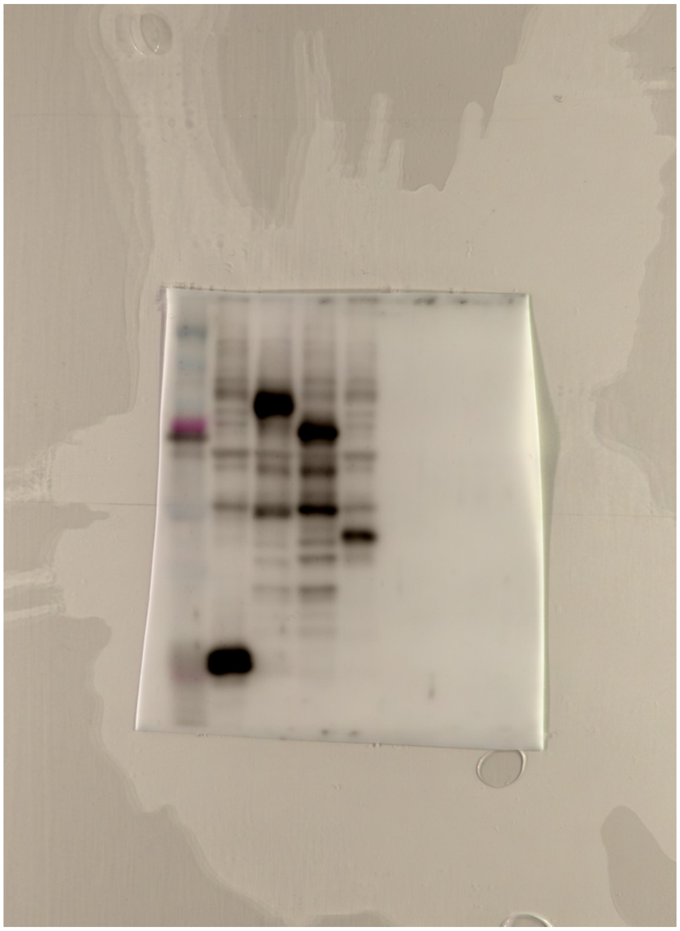

JPG file

Figure 1B

Input_LATS2

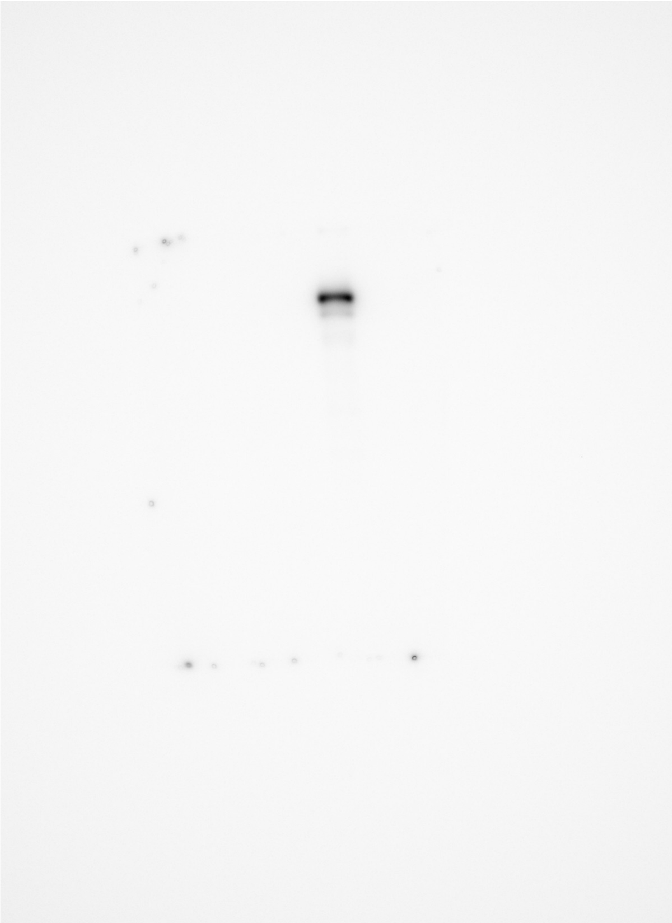

.TIF file

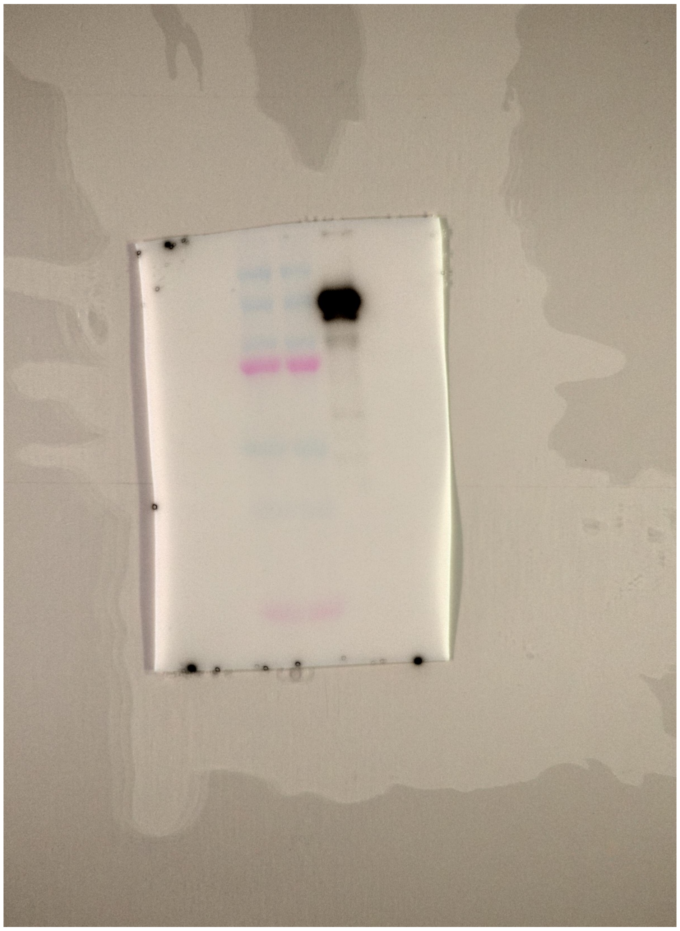

JPG file

Figure 1C

IP-Myc_LIMD1 (GFP)

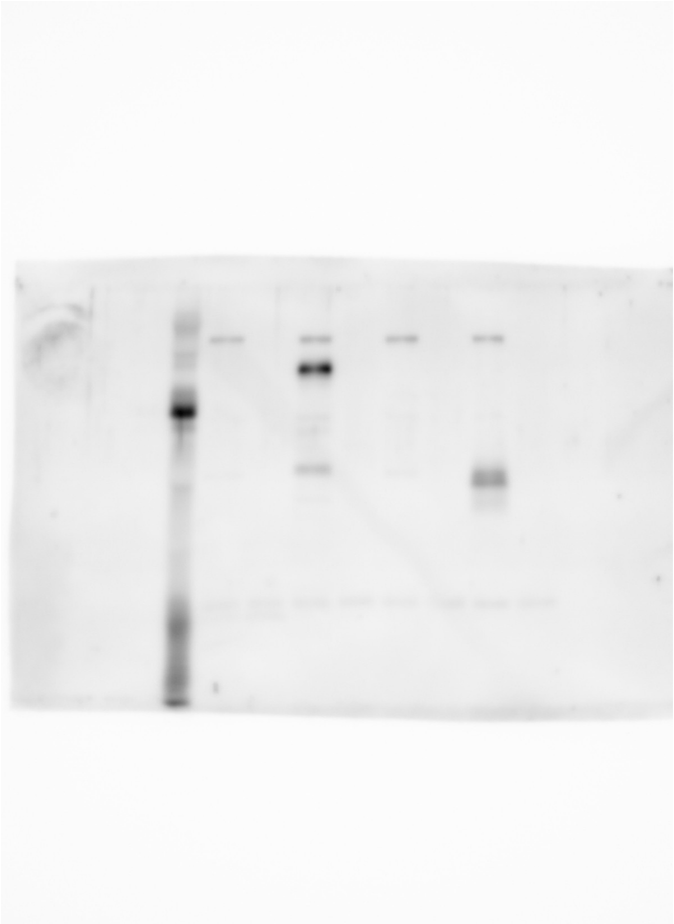

.TIF file

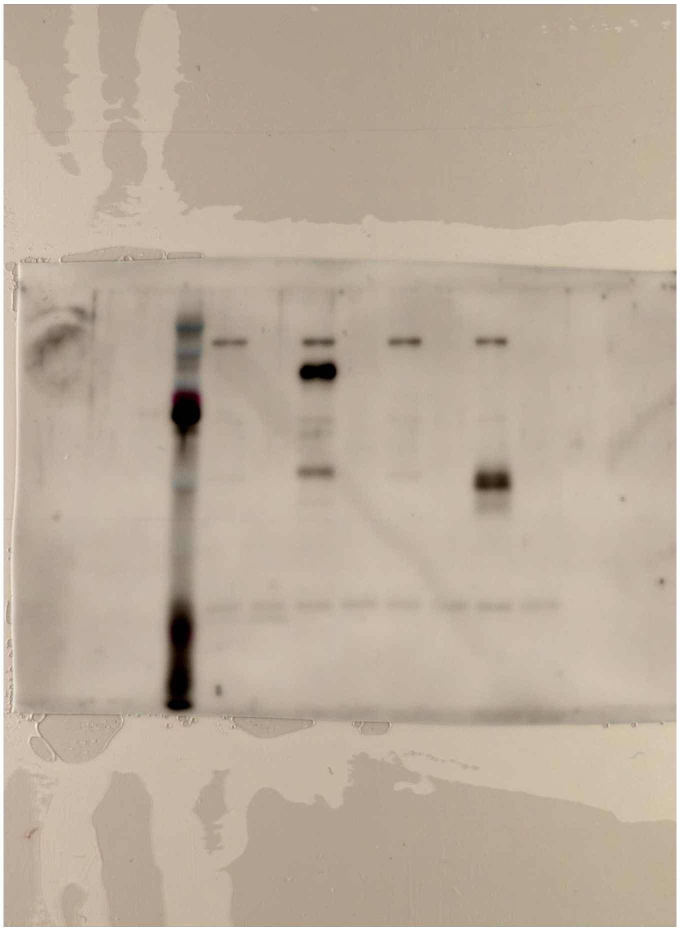

JPG file

Figure 1C

IP-Myc_LATS1

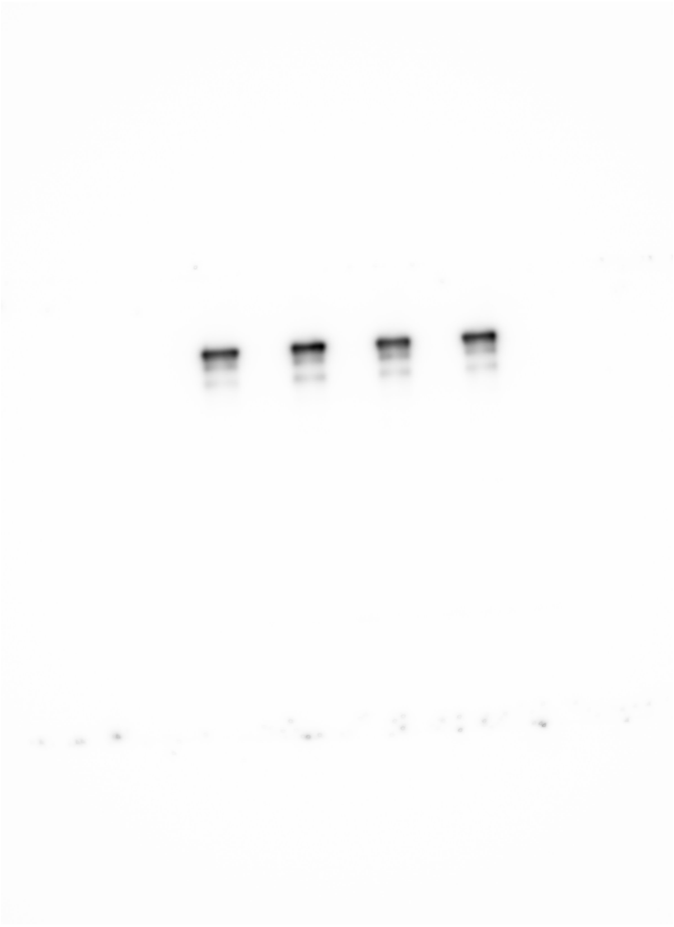

.TIF file

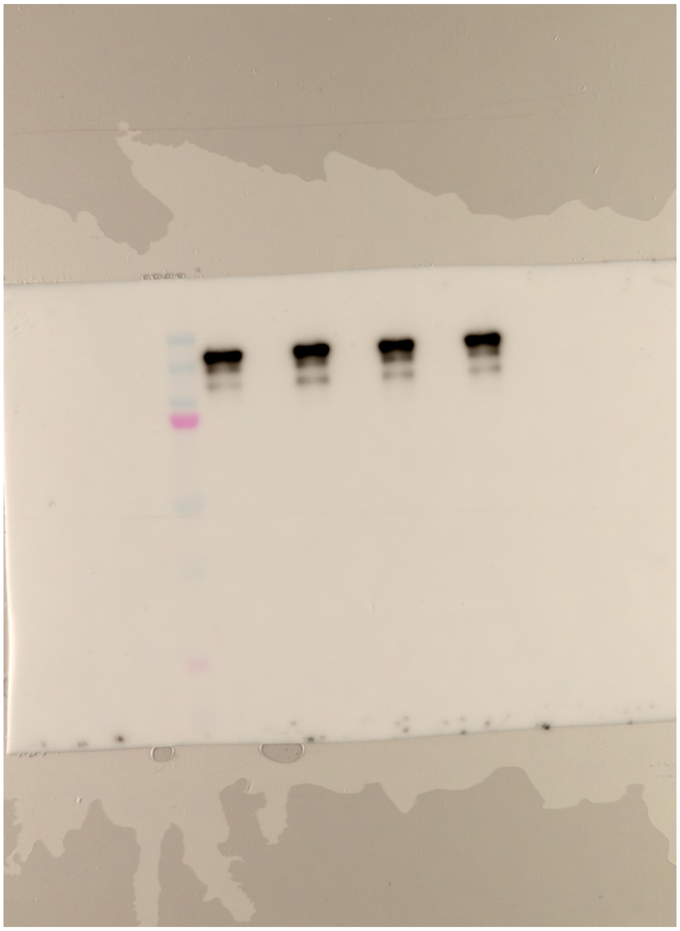

JPG file

Figure 1C

Input_LIMD1 (GFP)

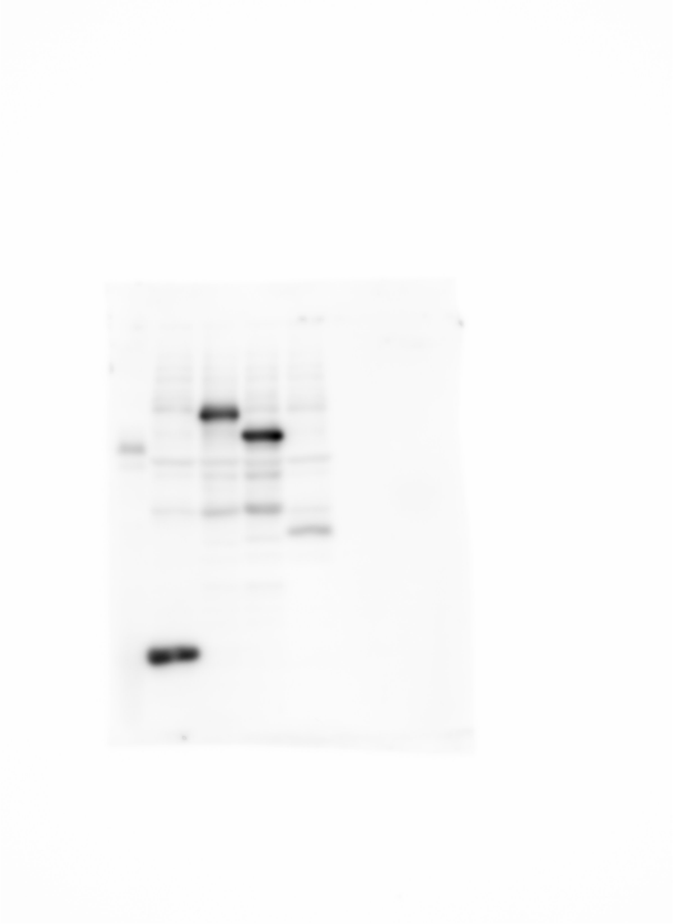

.TIF file

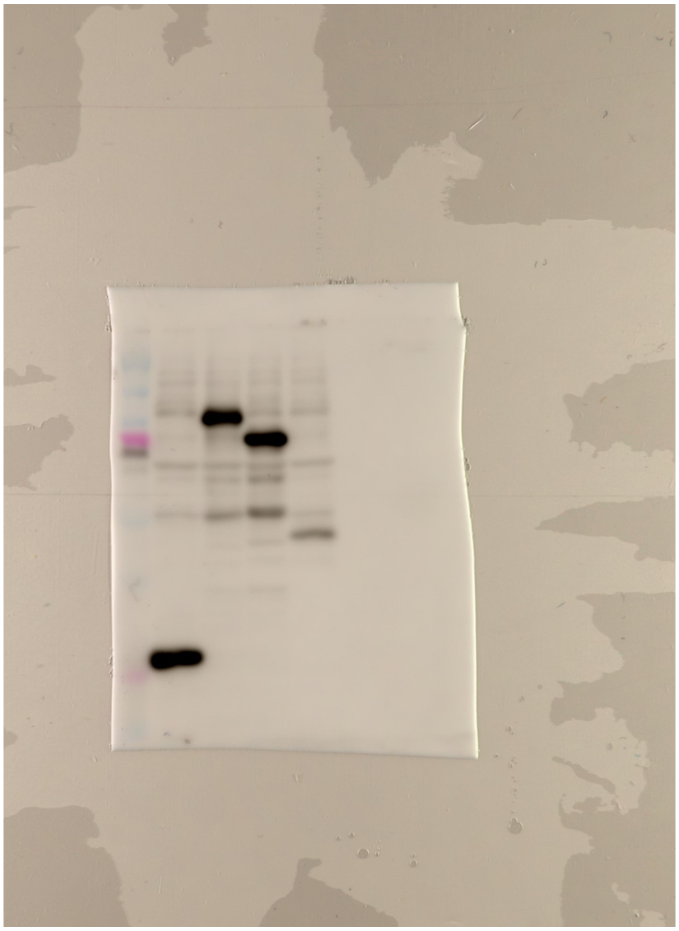

JPG file

Figure 1C

Input_LATS1

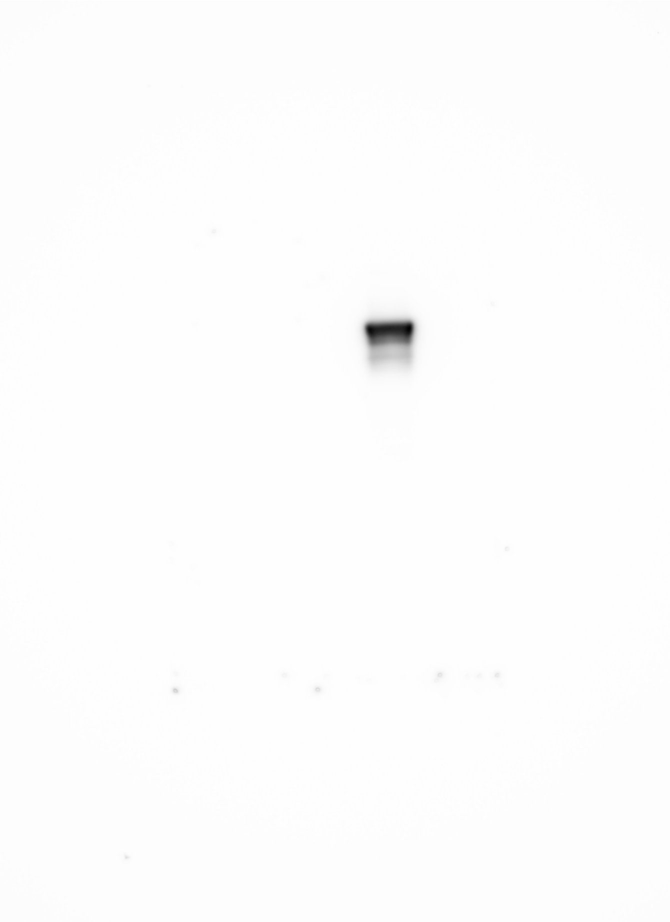

.TIF file

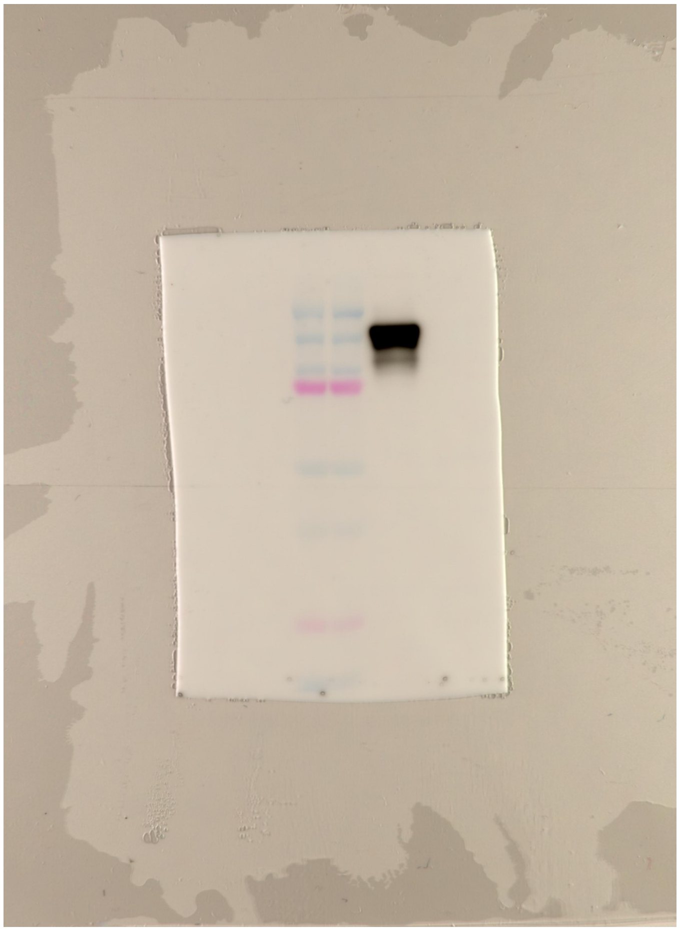

JPG file

Figure 3D

IP-LIMD1_LATS2

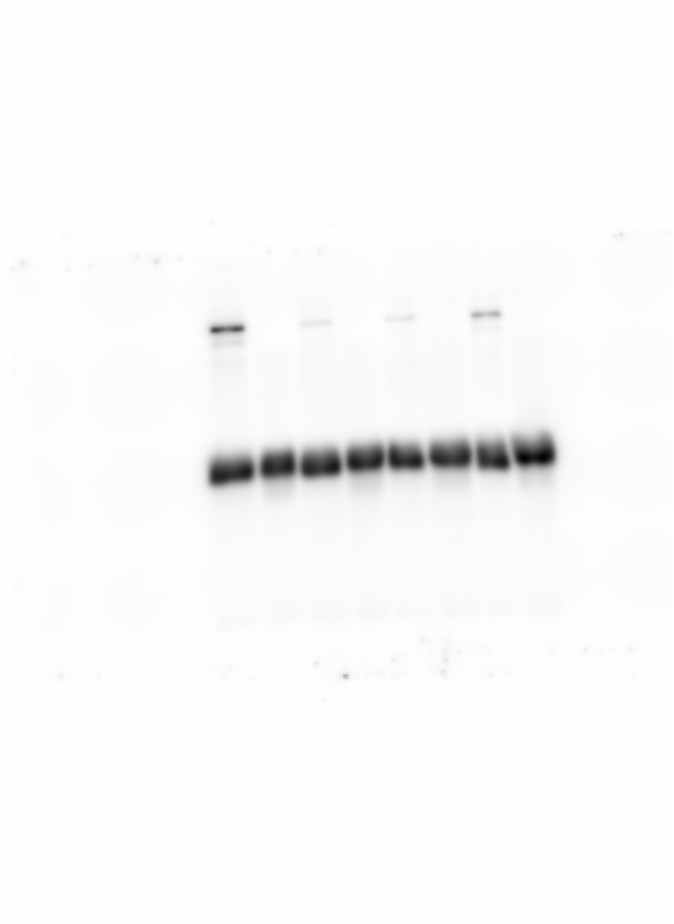

.TIF file

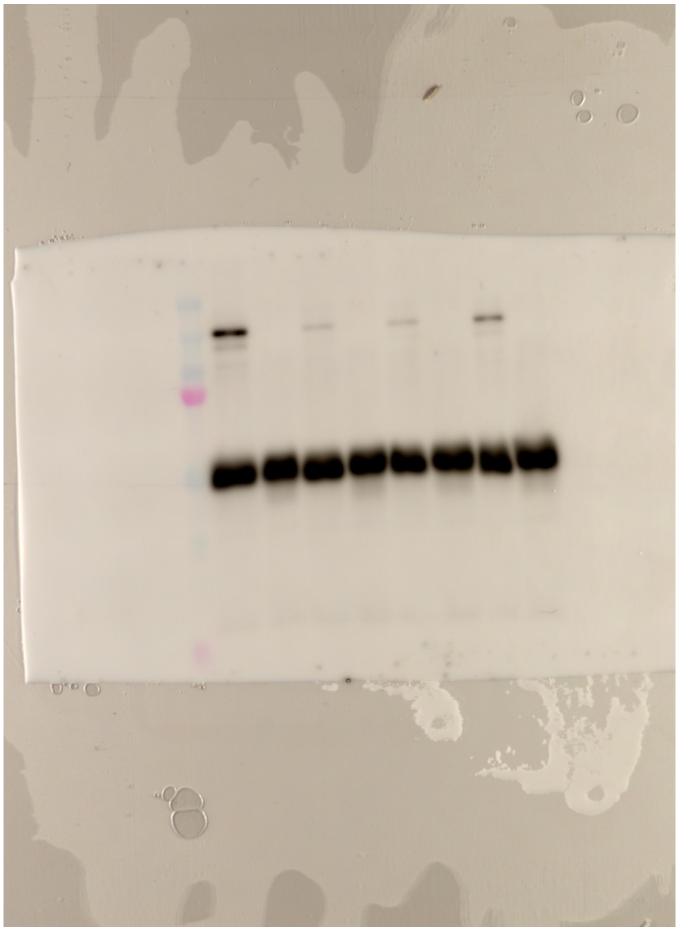

JPG file

Figure 3D

IP-LIMD1_LIMD1 (V5)

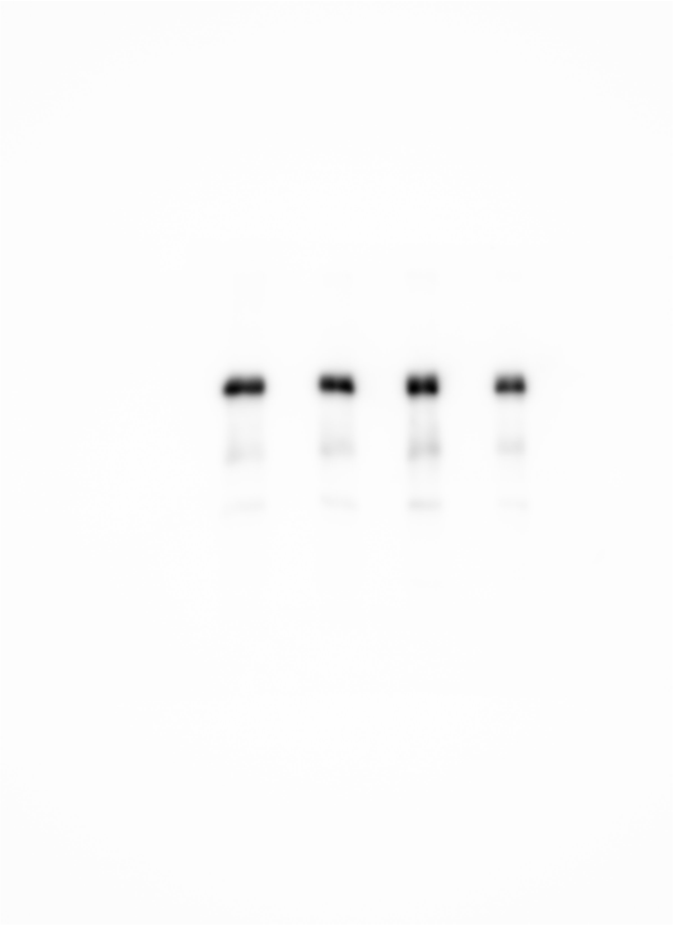

.TIF file

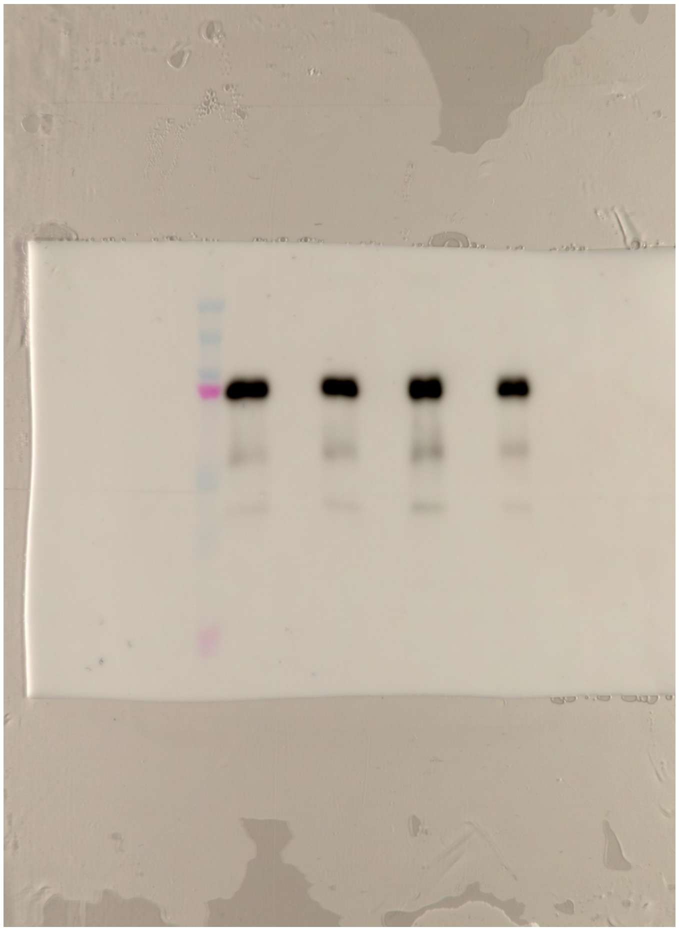

JPG file

Figure 3D

Input_LATS2

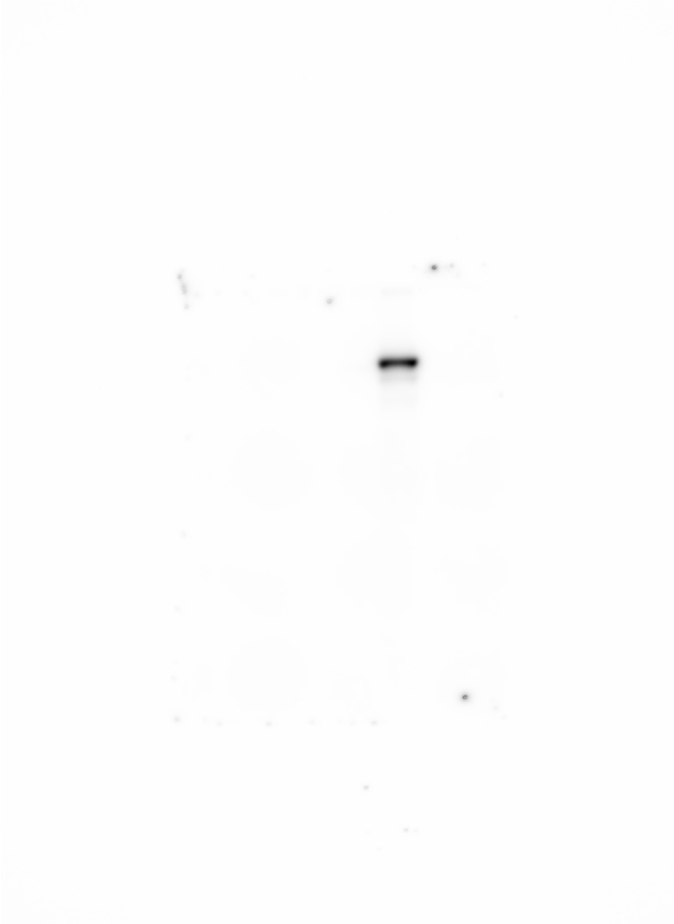

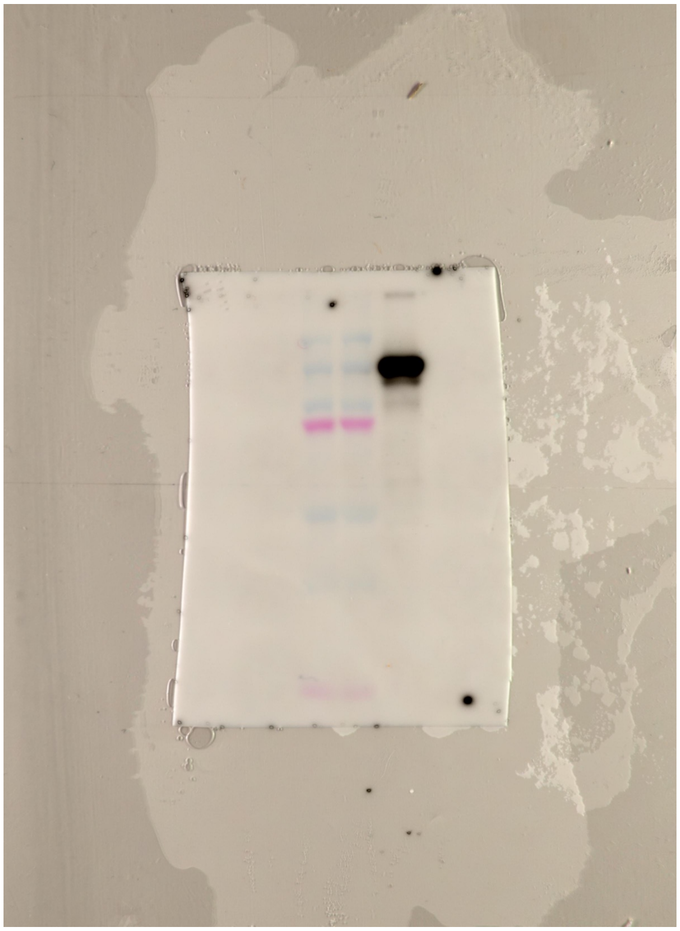

.TIF file

JPG file

Figure 3D

Input_LIMD1 (V5)

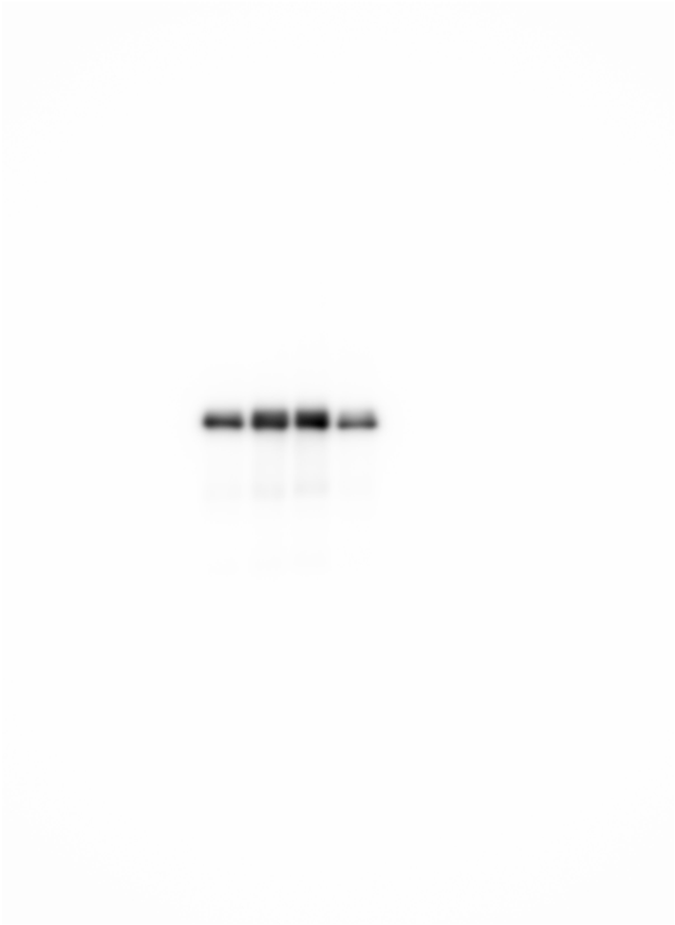

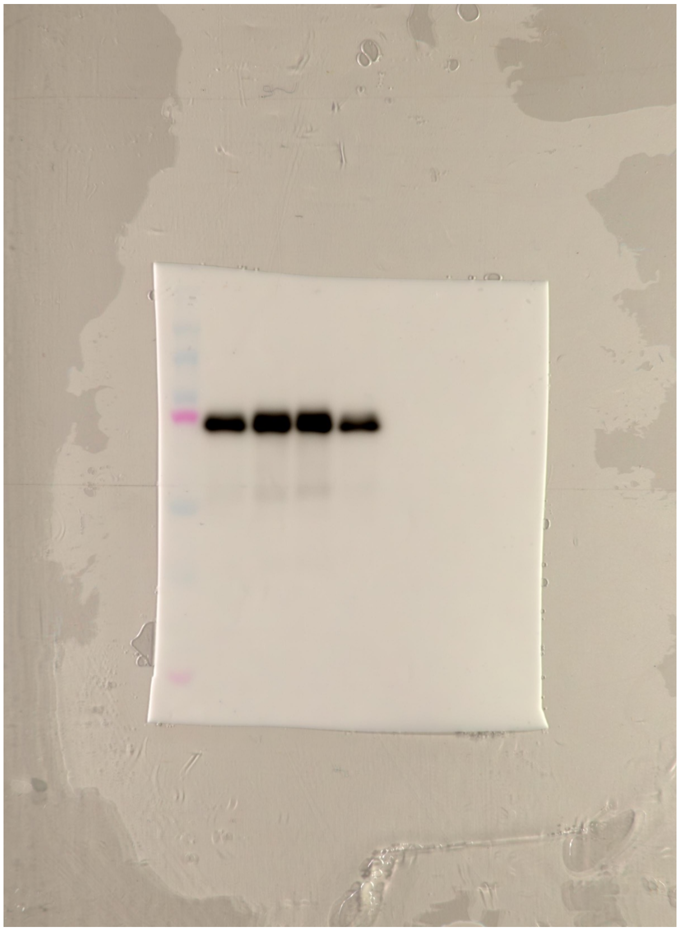

.TIF file

JPG file

Figure 3E

IP-Myc_LIMD1

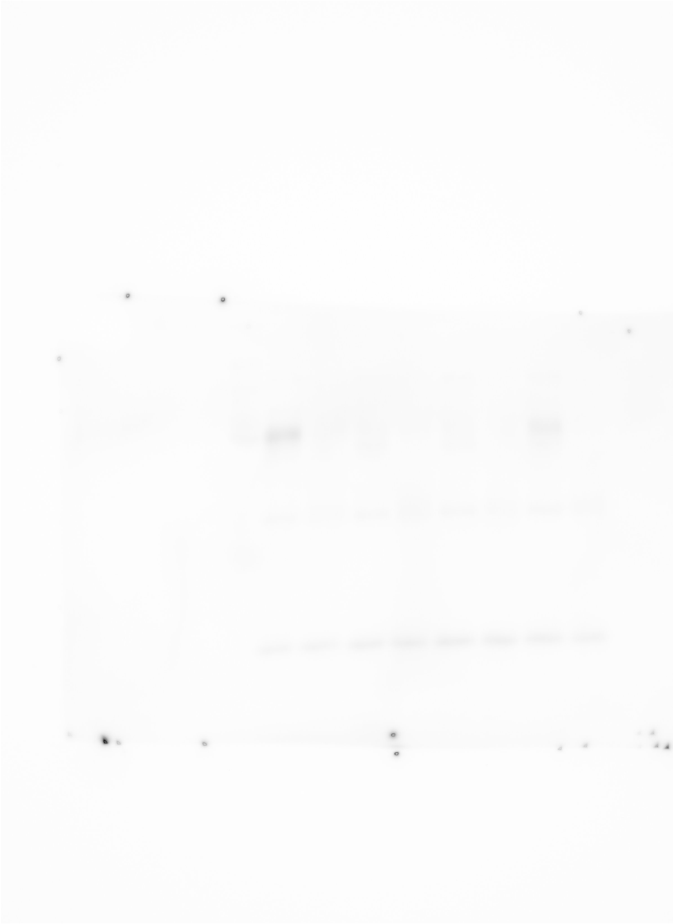

.TIF file

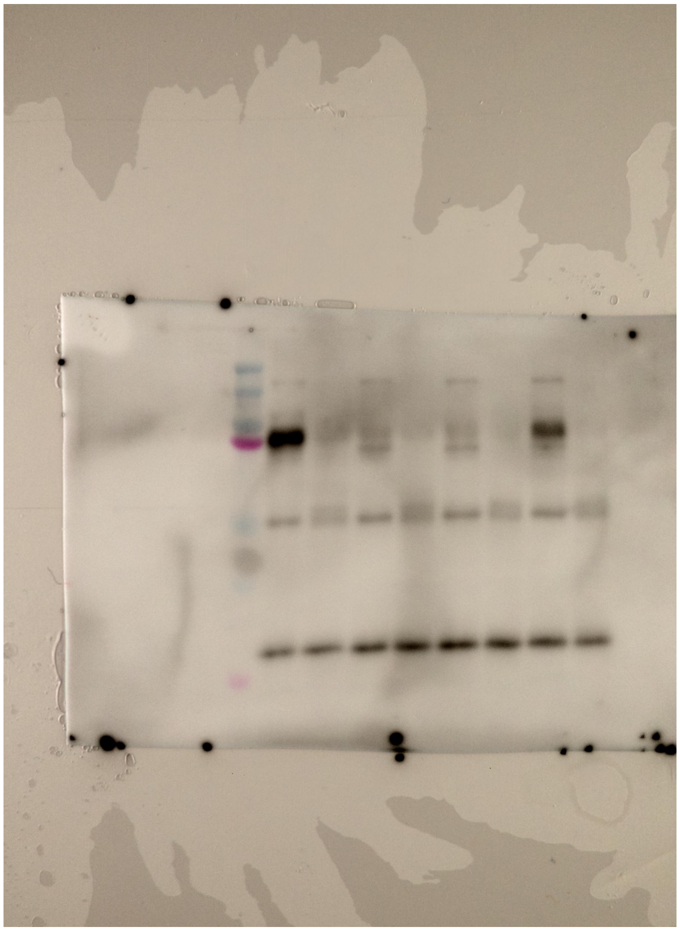

JPG file

Figure 3E

IP-Myc_LATS1

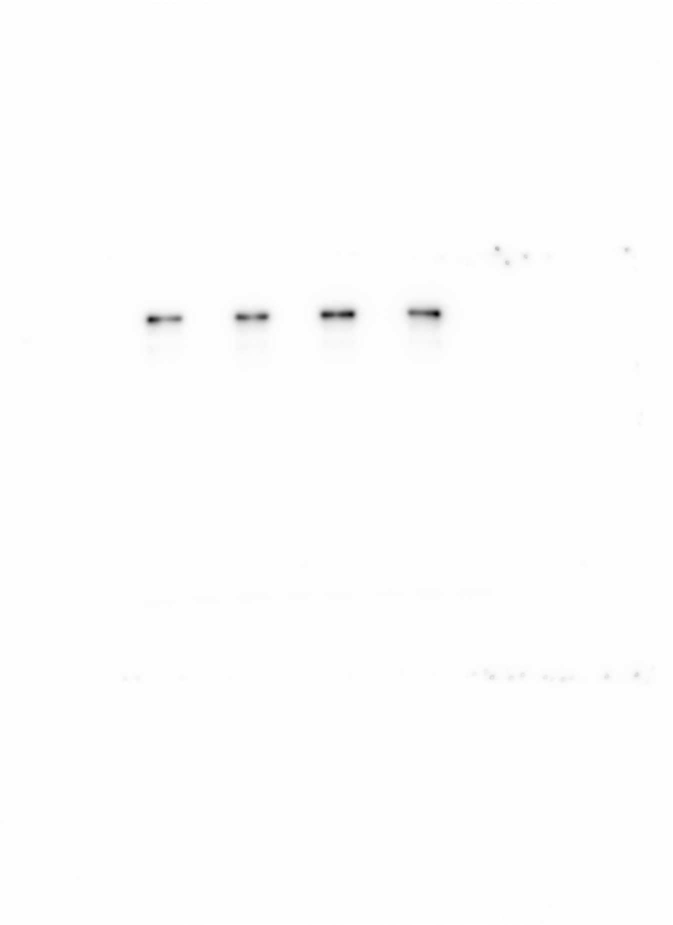

.TIF file

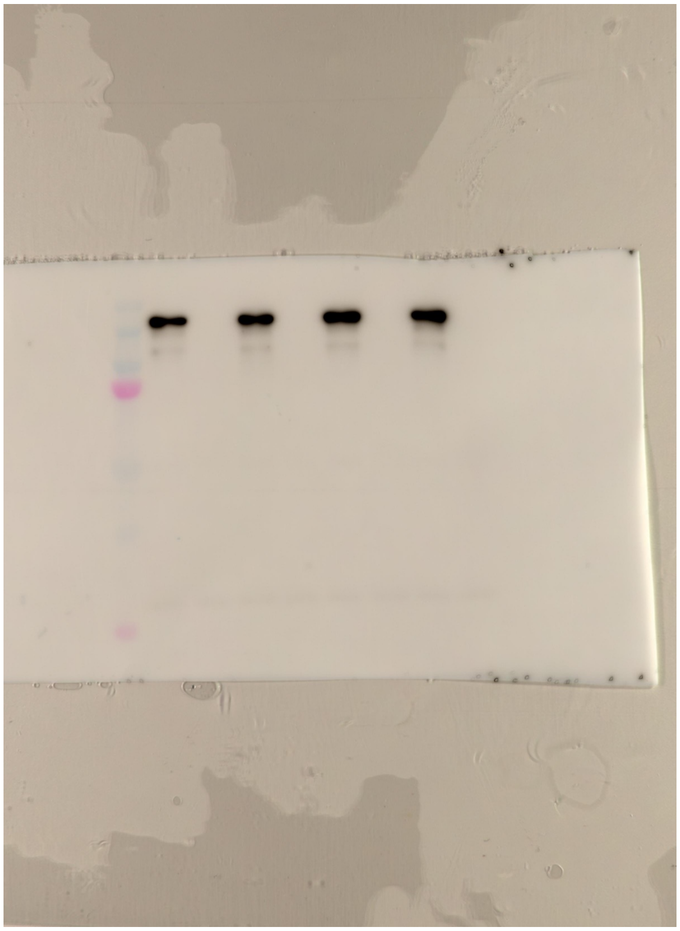

JPG file

Figure 3E

Input_LIMD1

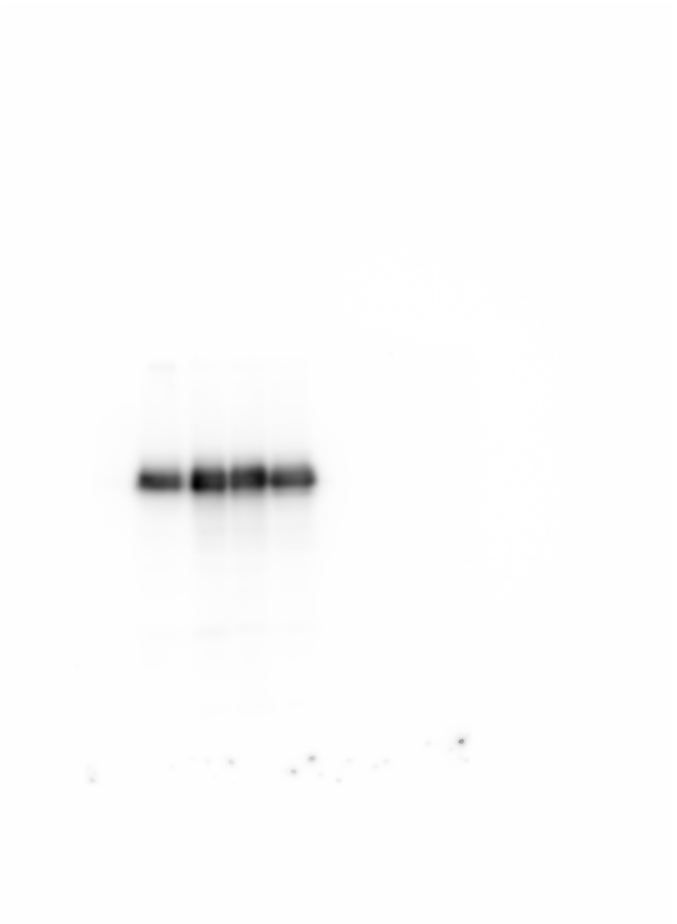

.TIF file

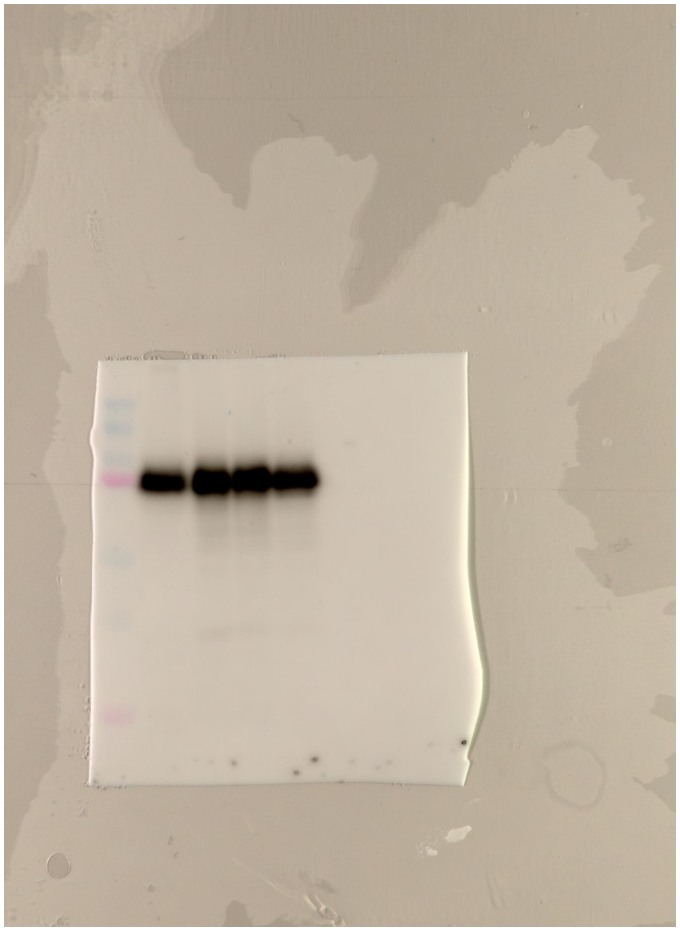

JPG file

Figure 3E

Input_LIMD1

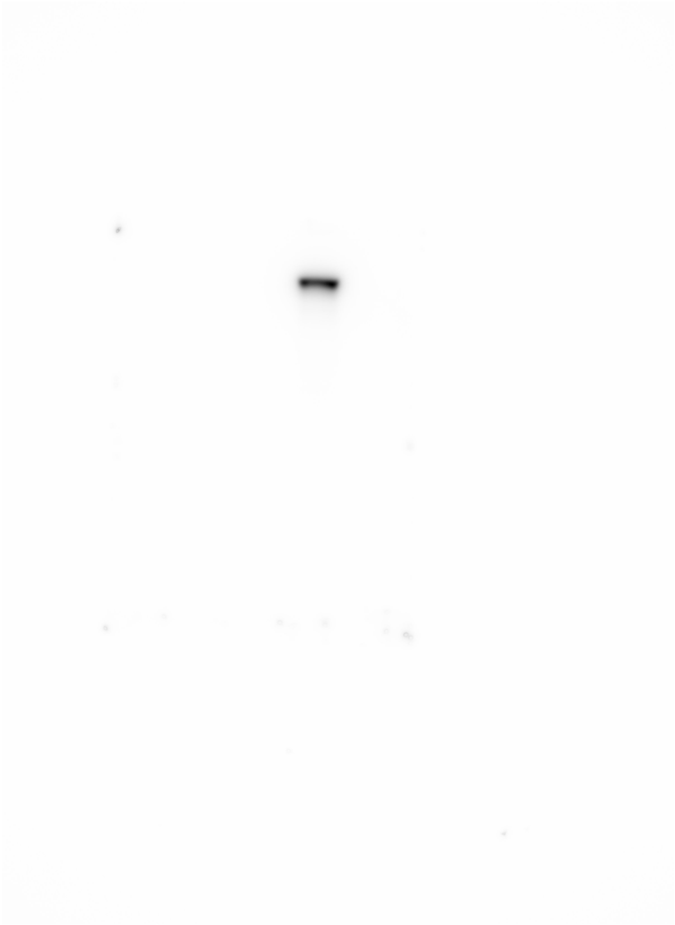

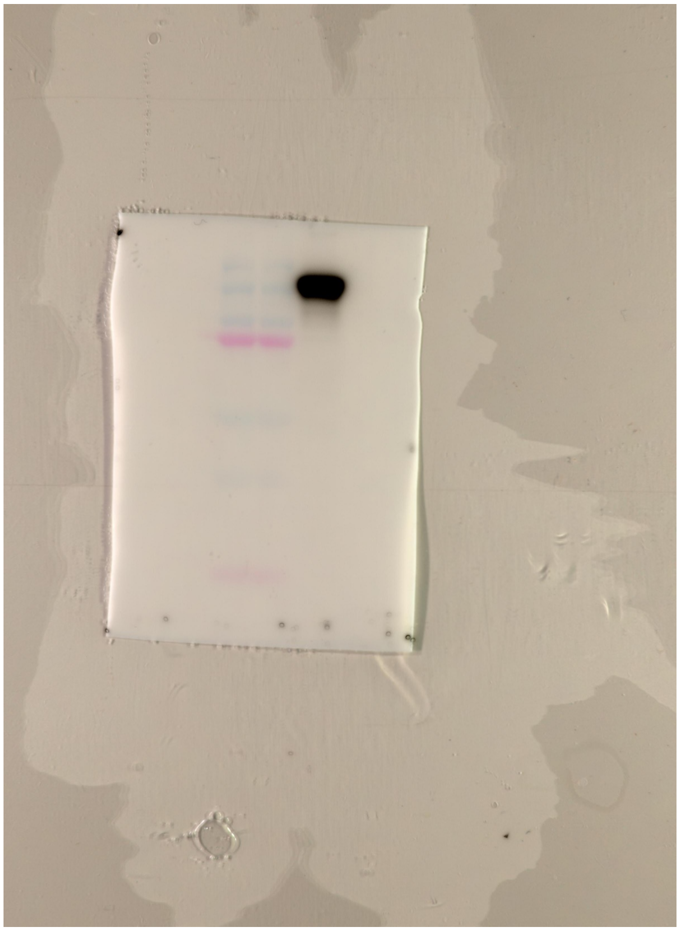

.TIF file **JPG file**

Figure 4D

IP-FLAG_LATS2 (FLAG)

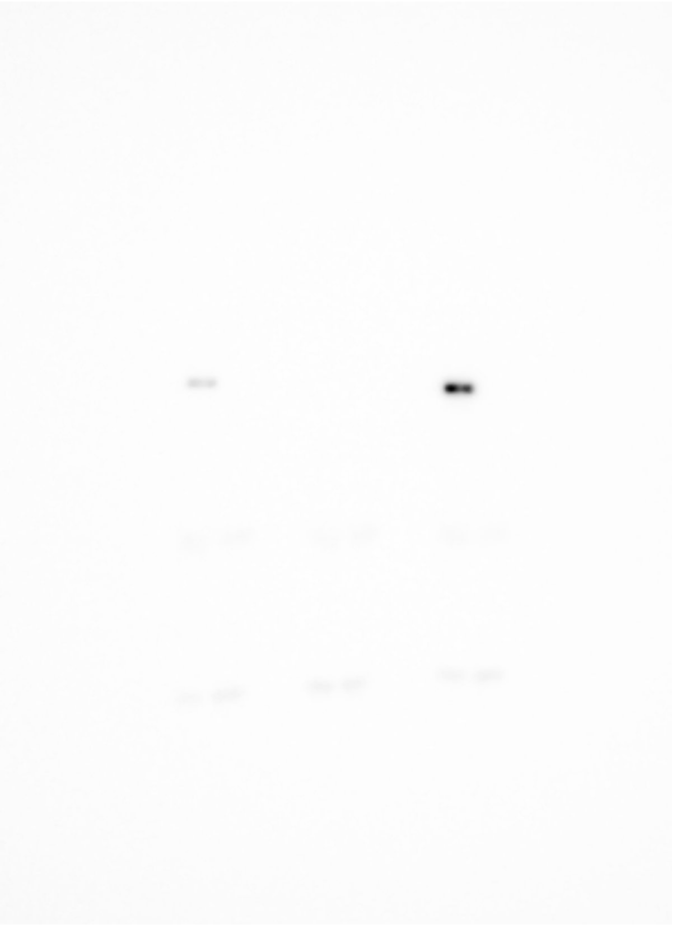

.TIF file

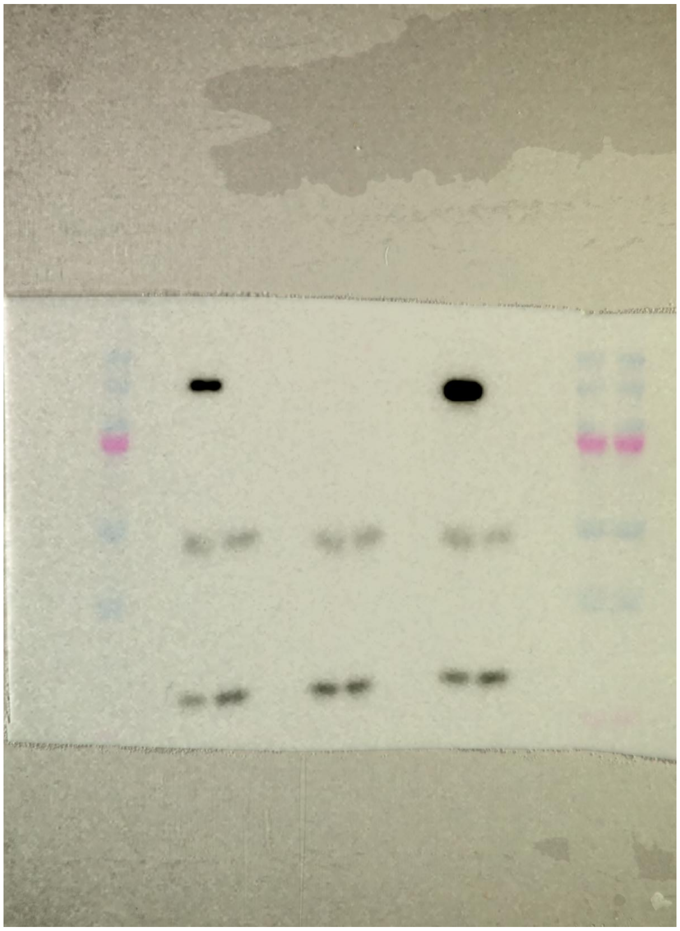

JPG file

Figure 4D

IP-FLAG_LIMD1 (V5)

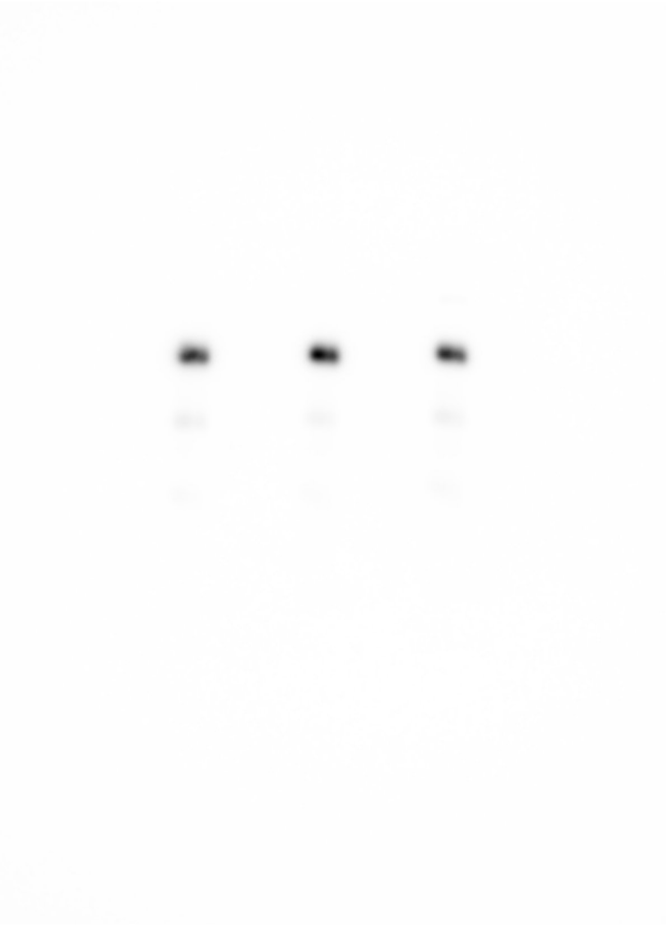

.TIF file

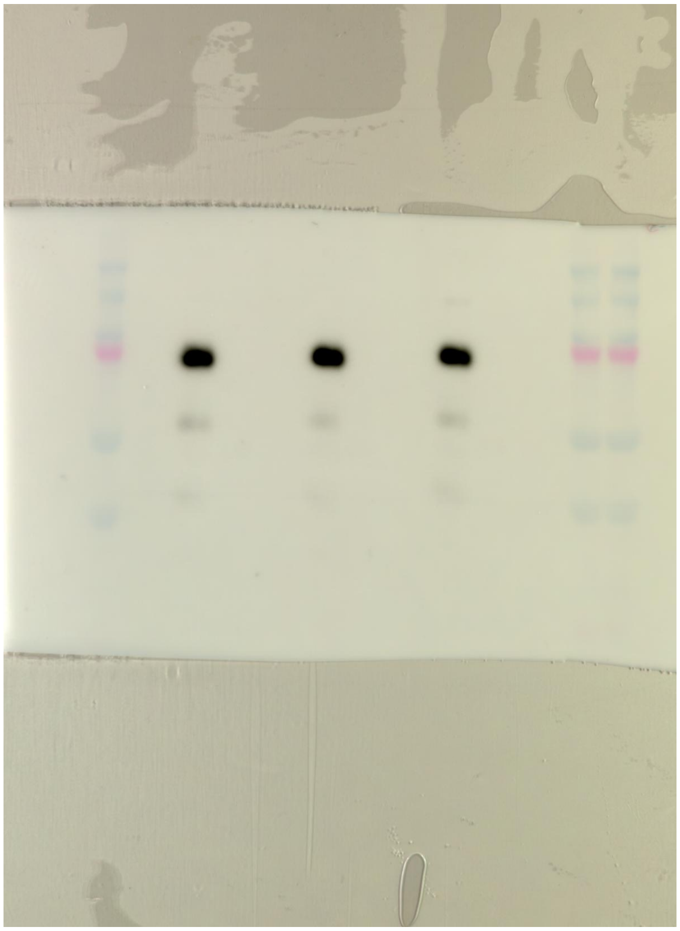

JPG file

Figure 4D

Input_LATS2 (FLAG)

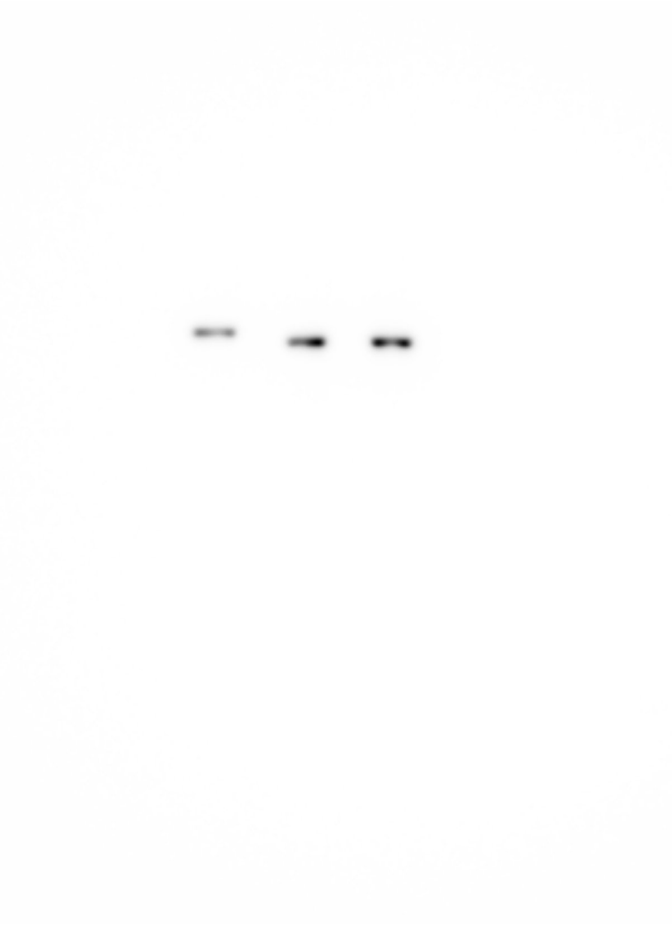

.TIF file

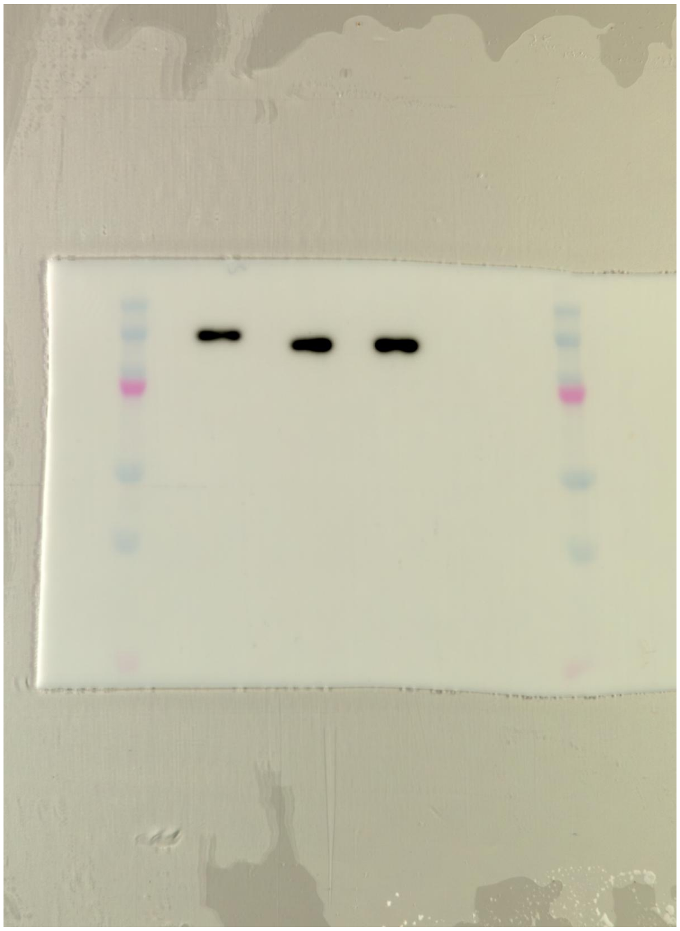

JPG file

Figure 4D

Input_LIMD1 (V5)

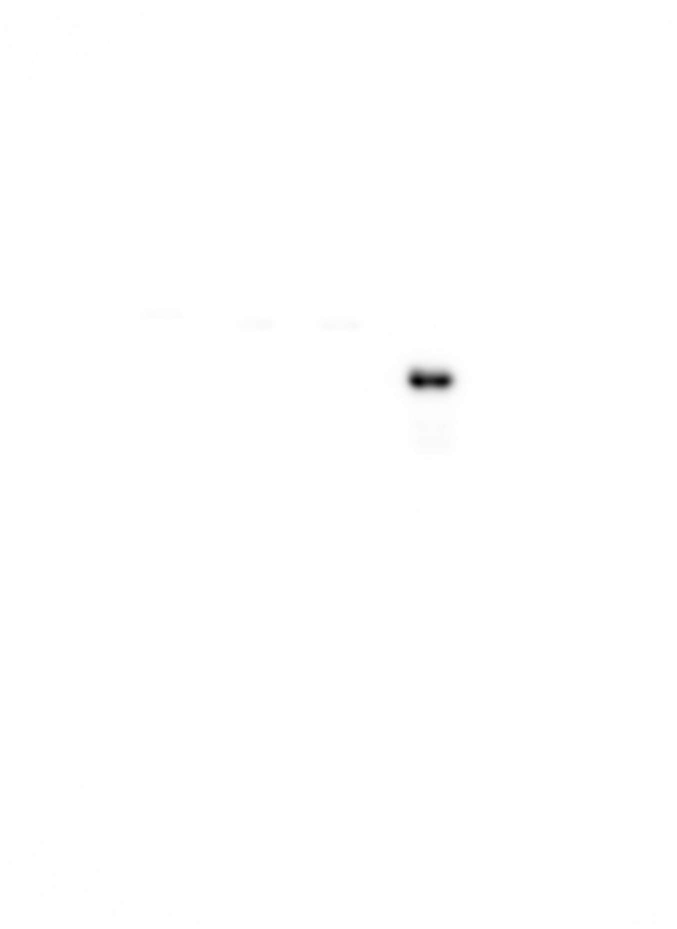

.TIF file

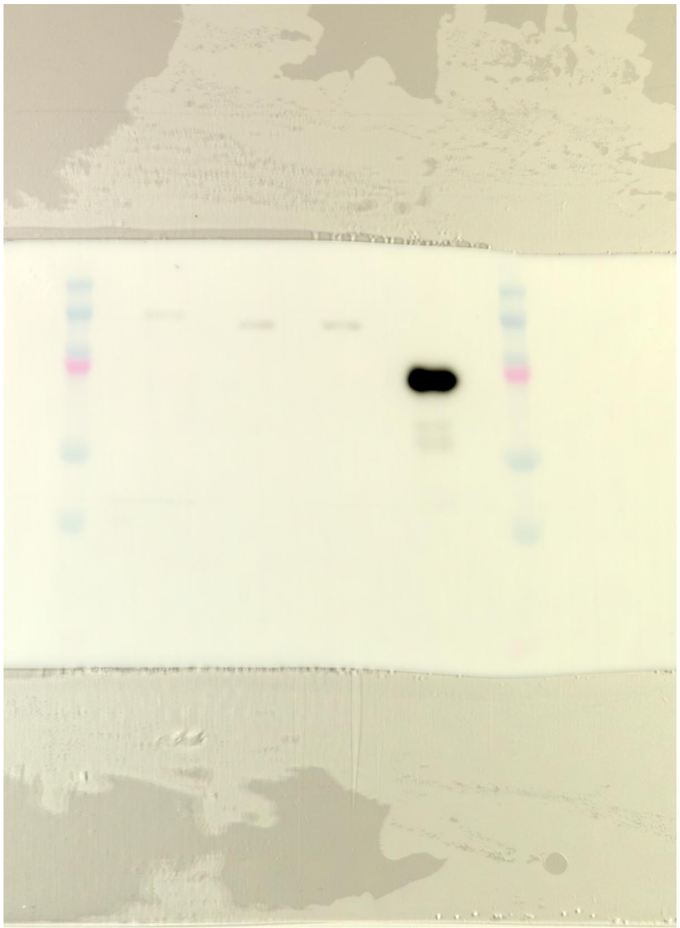

JPG file

Figure 4E

IP-LIMD1_LATS1 (Myc)

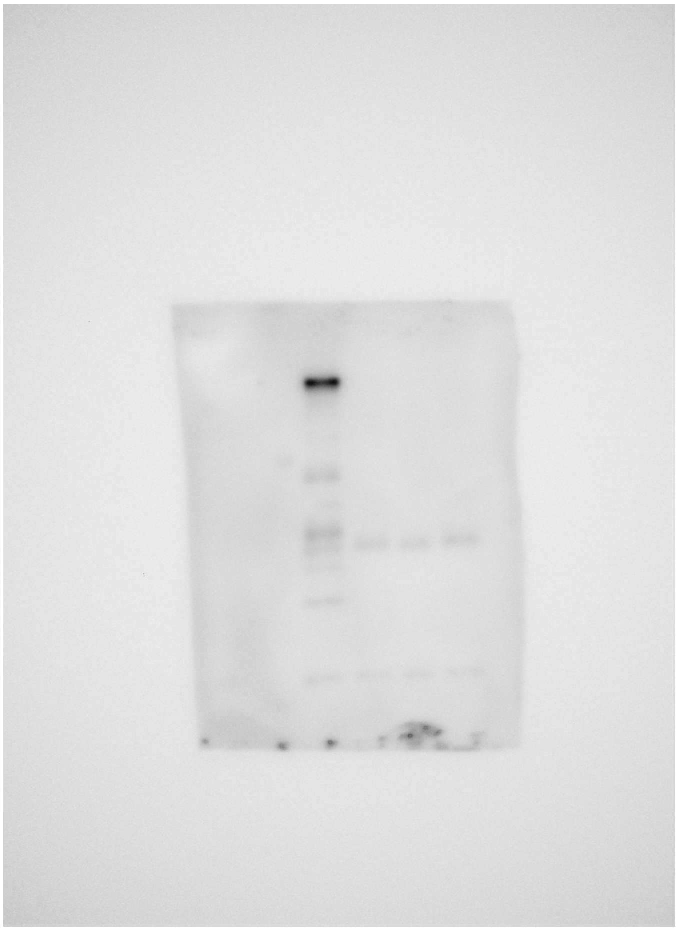

.TIF file

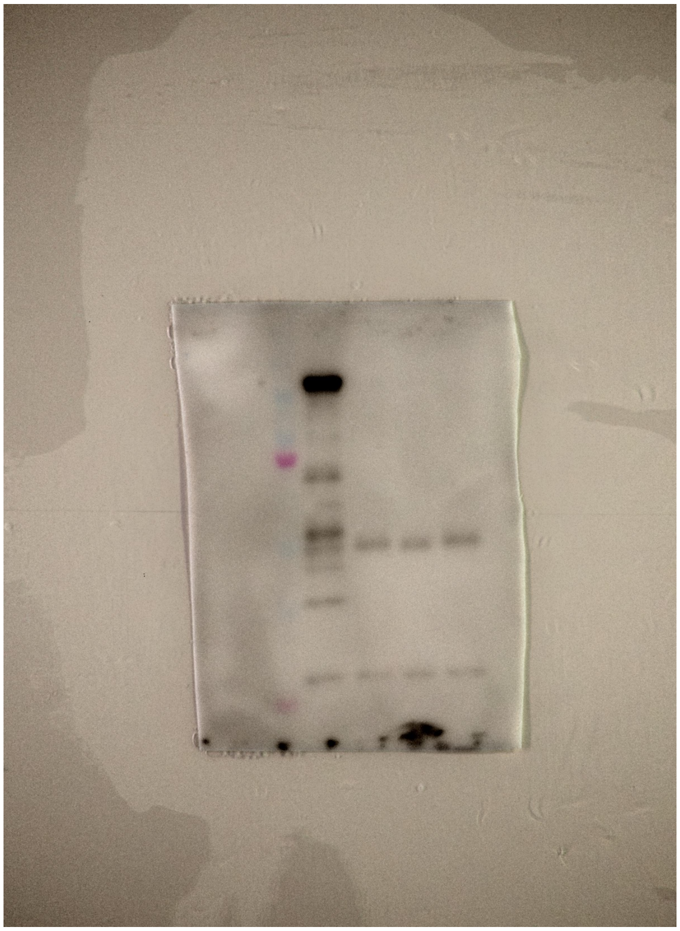

JPG file

Figure 4E

IP-LIMD1_LIMD1 (V5)

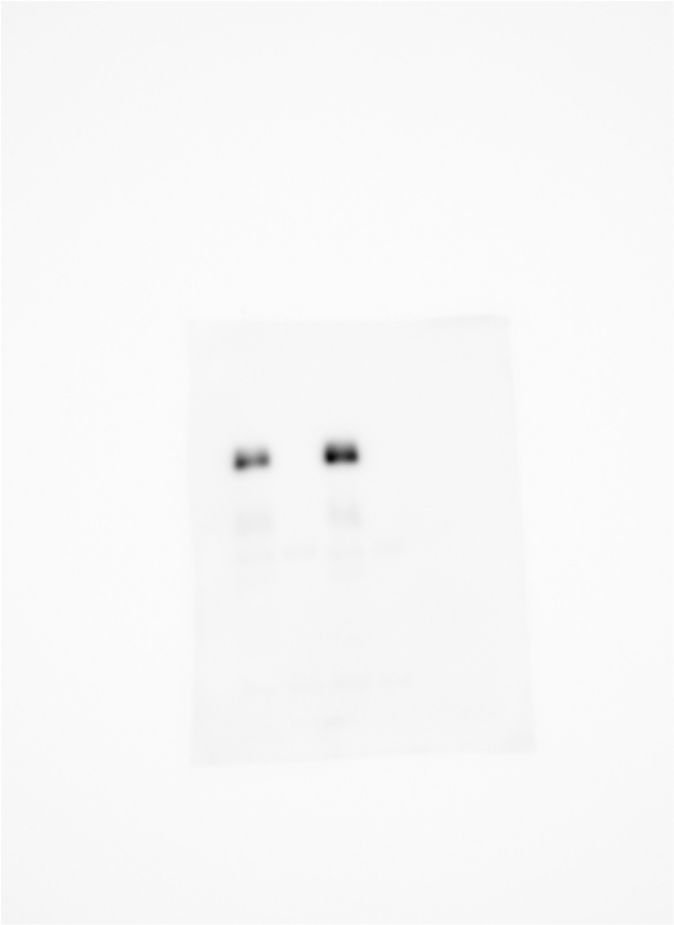

.TIF file

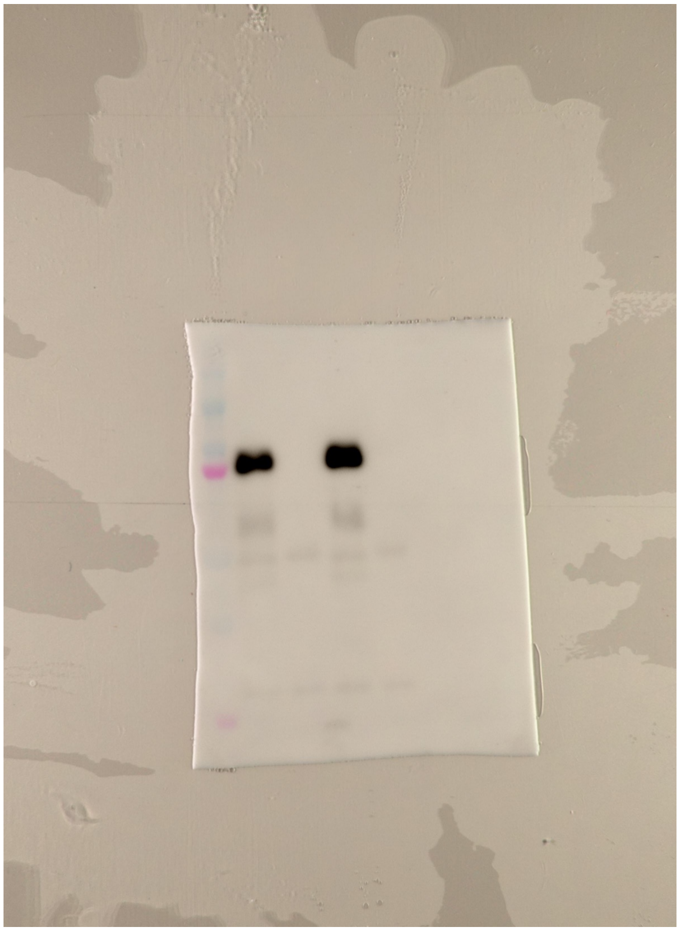

JPG file

Figure 4E

Input_LATS1 (Myc)

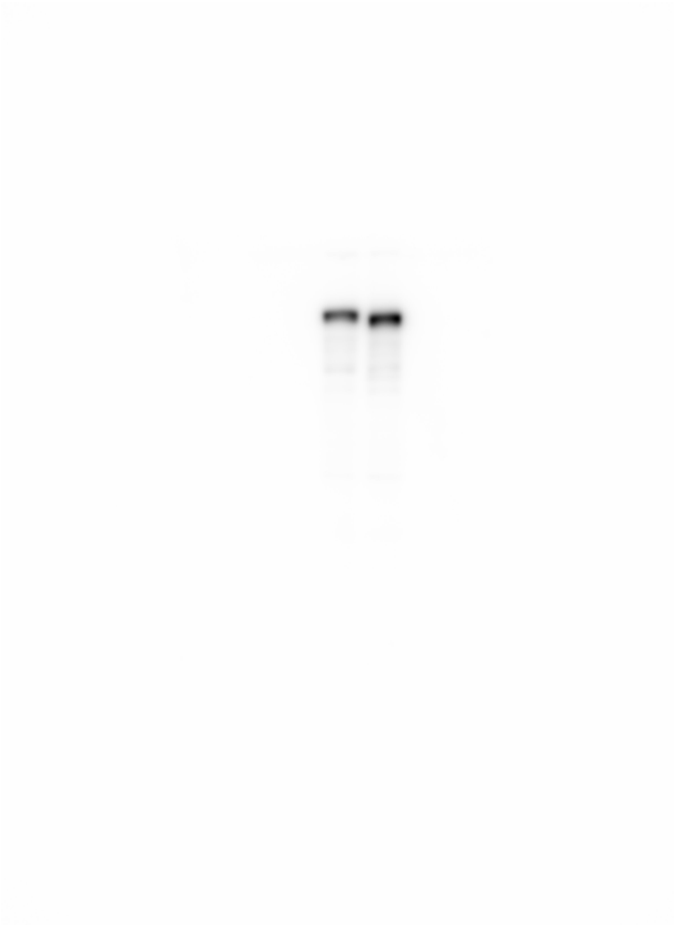

.TIF file

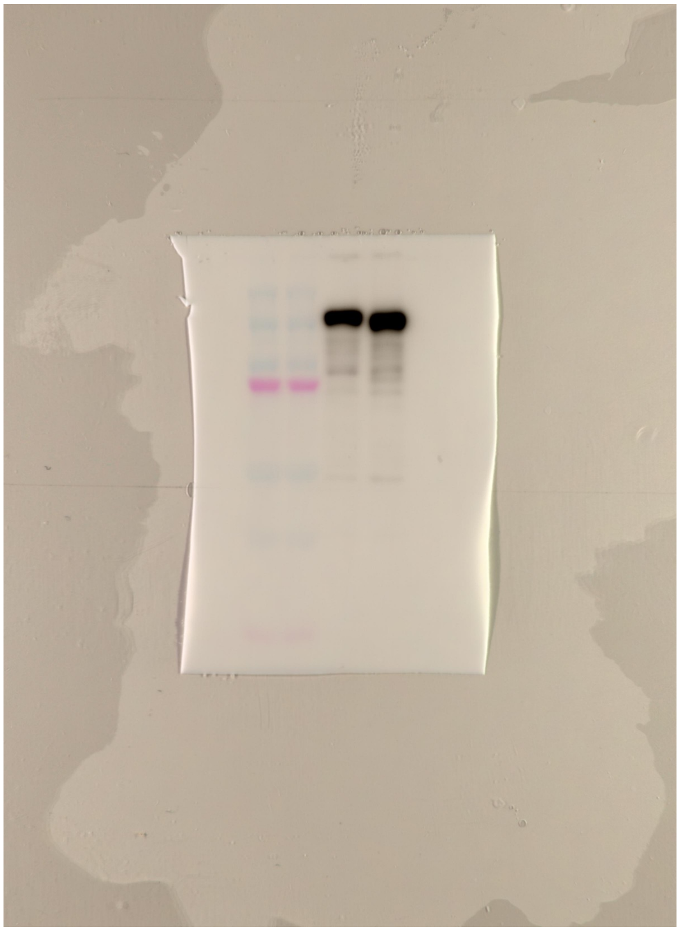

JPG file

Figure 4E

Input_LIMD1 (V5)

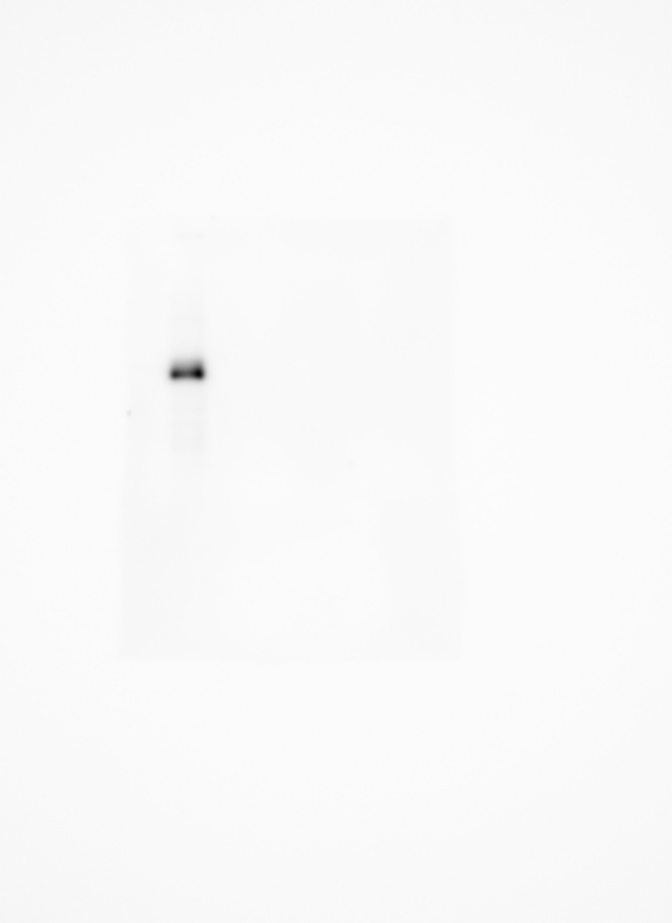

.TIF file

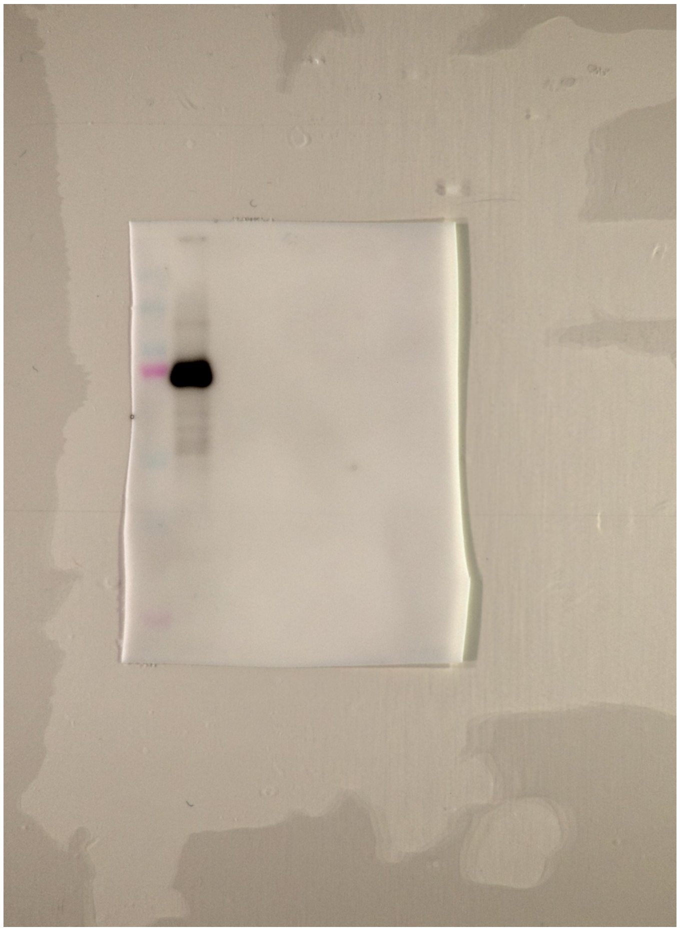

JPG file

Figure 5A

IP-LIMD1_LATS2 (GFP)

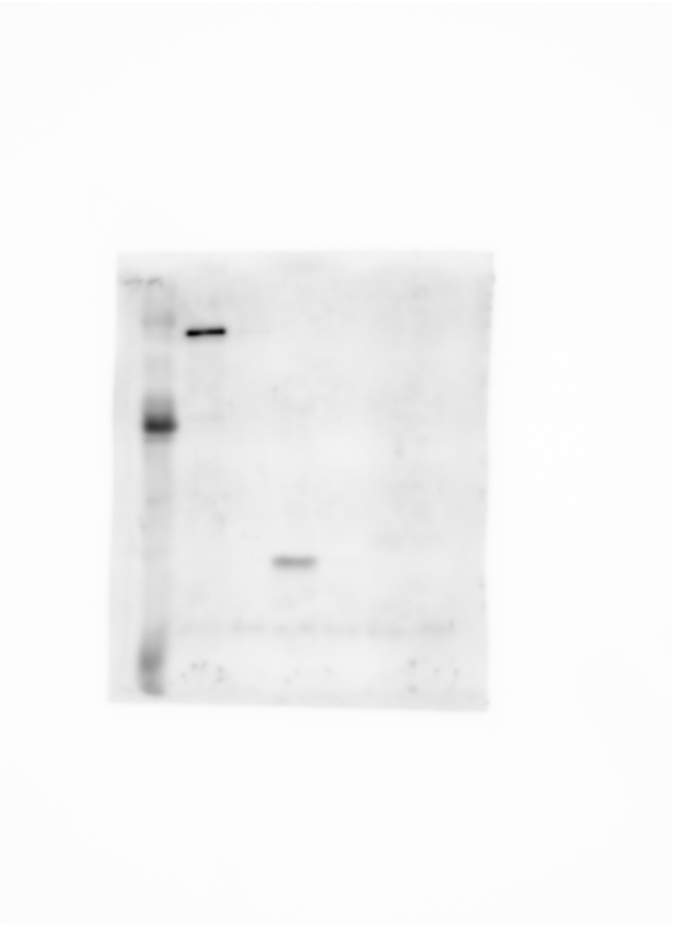

.TIF file

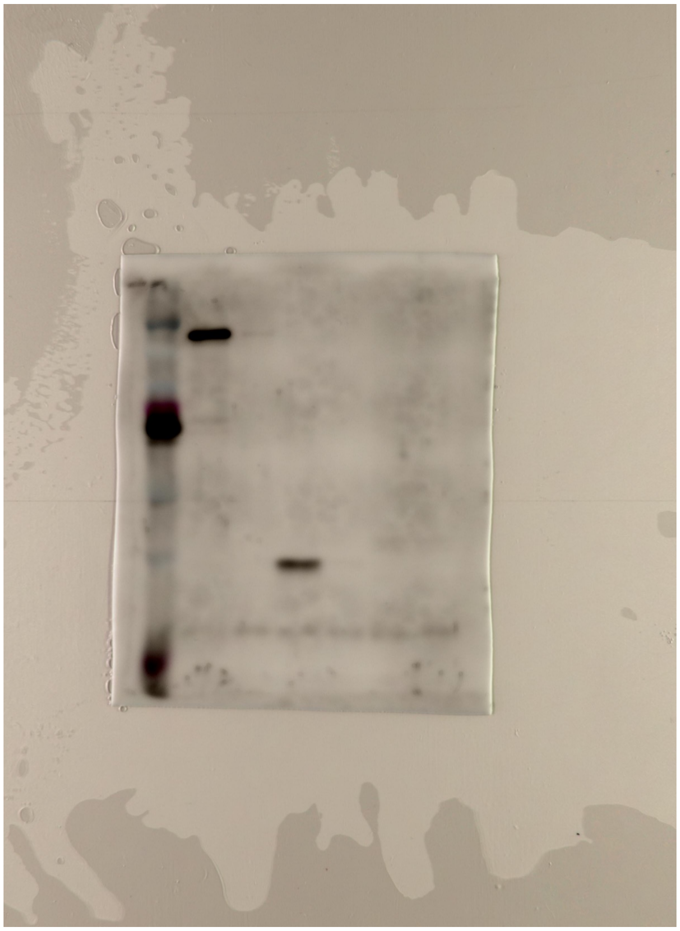

JPG file

Figure 5A

IP-LIMD1_LIMD1 (V5)

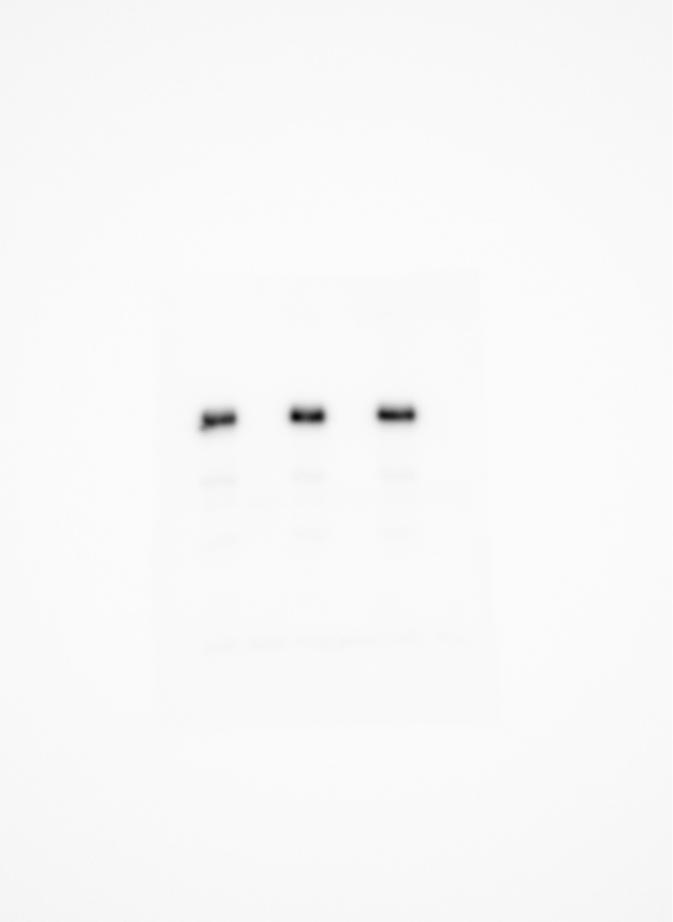

.TIF file

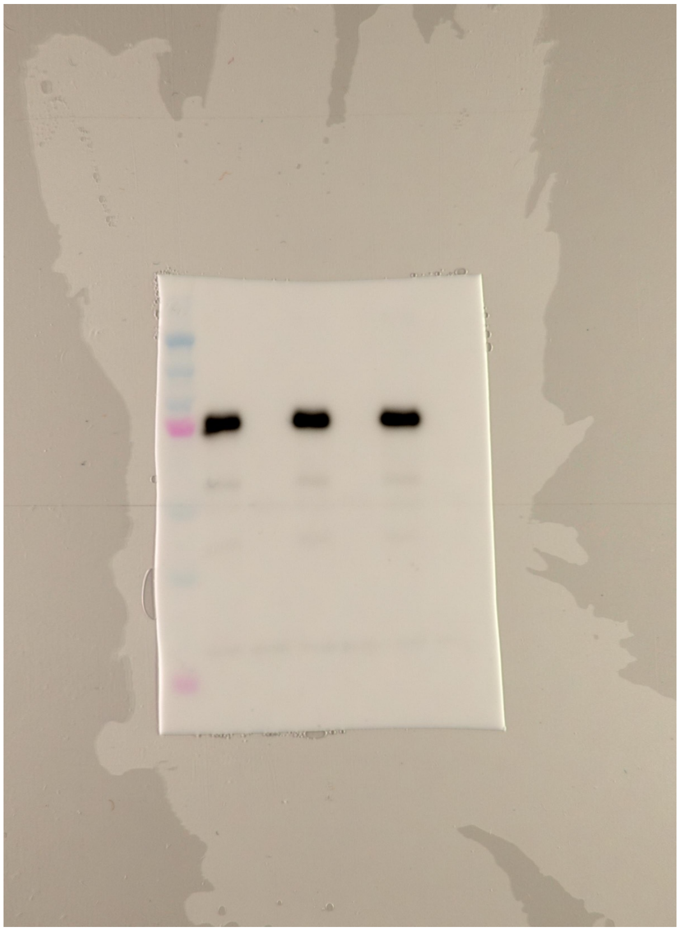

JPG file

Figure 5A

Input_LATS2 (GFP)

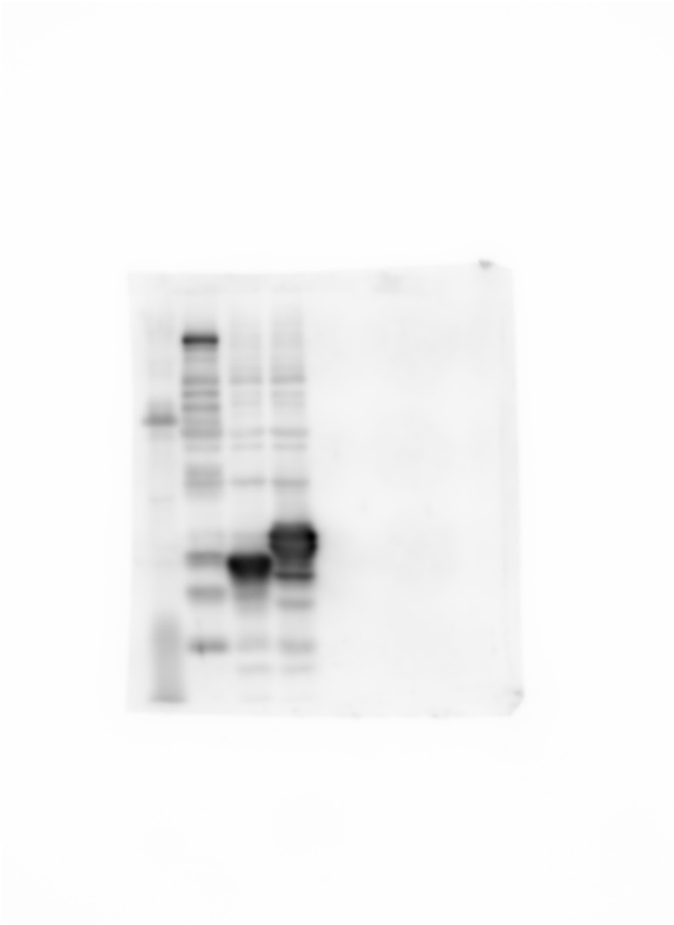

.TIF file

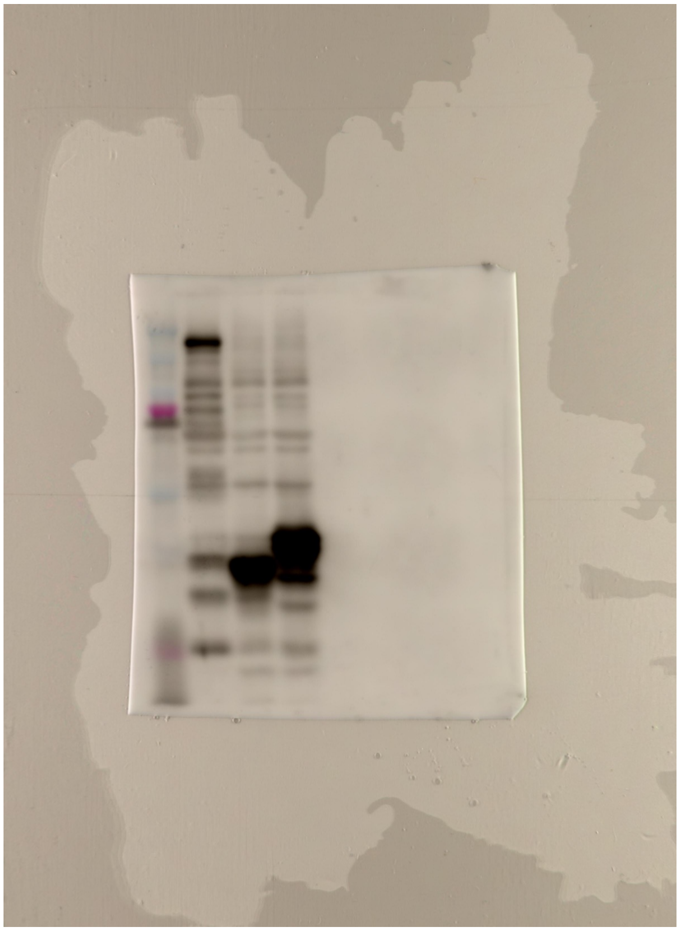

JPG file

Figure 5A

Input_LIMD1 (V5)

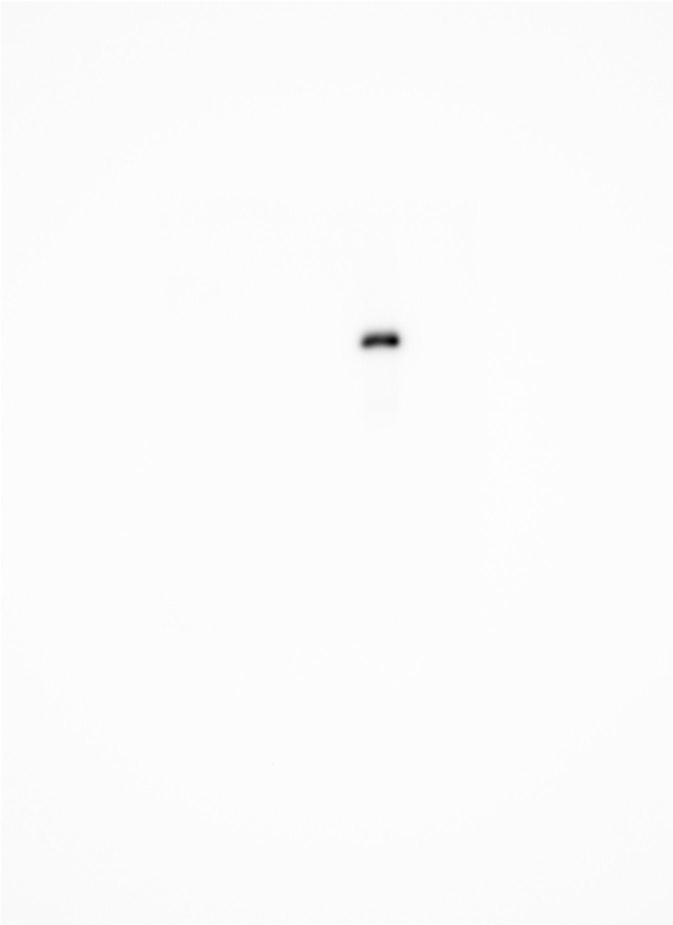

.TIF file

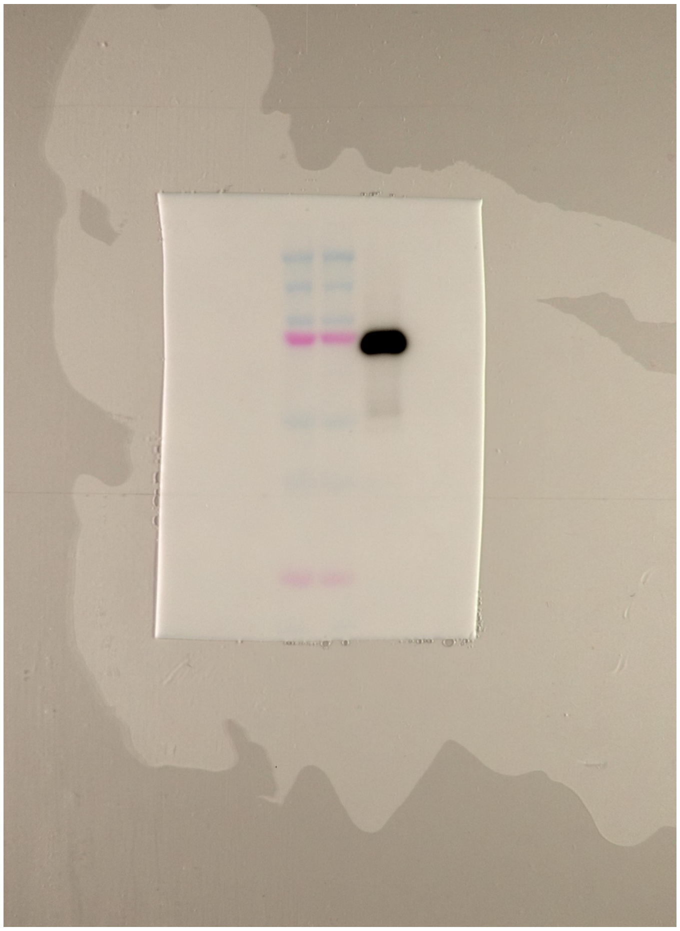

JPG file
