# Supplementary figures and images for: Regulation of tension-dependent localization of LATS1 and LATS2 to adherens junctions
Source: PLoS One. 2026 Feb 2;21(2):e0342107. doi: 10.1371/journal.pone.0342107 (PMC12863670; doi:10.1371/journal.pone.0342107)

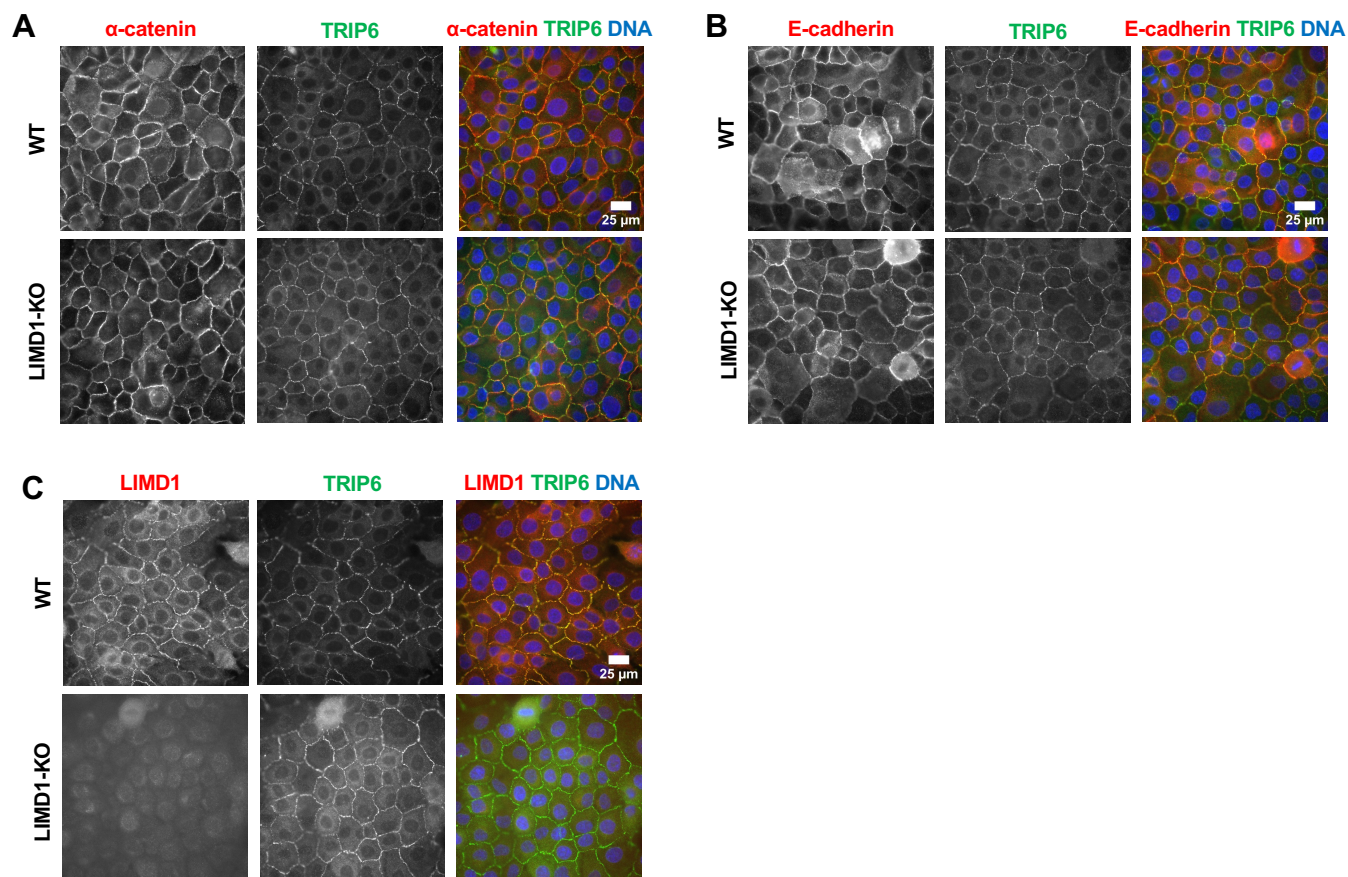

**S1 Fig.**

Supplement: S2 Fig — Wild-type (WT) and LIMD1 knockout (LIMD1-KO) MCF10A cells were stained using anti-TRIP6 antibody together with (A) anti-alpha-E-Catenin, (B) anti-E-cadherin or (C) anti-LIMD1 antibody as indicated. Merged images show alpha-E-Catenin/ E-cadherin/LIMD1 (red), TRIP6 (green) and DNA (blue). (PDF) [file pone.0342107.s002.pdf]
